# Marine-freshwater prokaryotic transitions require extensive changes in the predicted proteome

**DOI:** 10.1186/s40168-019-0731-5

**Published:** 2019-08-22

**Authors:** Pedro J. Cabello-Yeves, Francisco Rodriguez-Valera

**Affiliations:** 1 Evolutionary Genomics Group, Departamento de Producción Vegetal y Microbiología, Universidad Miguel Hernández, San Juan de Alicante, 03550 Alicante, Spain; 2 Moscow Institute of Physics and Technology, Dolgoprudny, 141701 Russia

**Keywords:** Isoelectric point, Marine-freshwater transition, Metaproteome, Basic shift, Electrostatic surface potential

## Abstract

**Background:**

The adaptation of a marine prokaryote to live in freshwater environments or vice versa is generally believed to be an unusual and evolutionary demanding process. However, the reasons are not obvious given the similarity of both kinds of habitats.

**Results:**

We have found major differences at the level of the predicted metaproteomes of marine and freshwater habitats with more acidic values of the isoelectric points (pI) in marine microbes. Furthermore, by comparing genomes of marine-freshwater phylogenetic relatives, we have found higher pI values (basic shift) in the freshwater ones. This difference was sharper in secreted > cytoplasmic > membrane proteins. The changes are concentrated on the surface of soluble proteins. It is also detectable at the level of total amino acid composition and involves similarly core and flexible genome- encoded proteins.

**Conclusions:**

The marked changes at the level of protein amino acid composition and pI provide a tool to predict the preferred habitat of a culture or a metagenome-assembled genome (MAG). The exact physiological explanation for such variations in the pIs and electrostatic surface potentials is not known yet. However, these changes might reflect differences in membrane bioenergetics derived from the absence of significant Na^+^ concentrations in most freshwater habitats. In any case, the changes in amino acid composition in most proteins imply that a long evolutionary time is required to adapt from one type of habitat to the other.

**Electronic supplementary material:**

The online version of this article (10.1186/s40168-019-0731-5) contains supplementary material, which is available to authorized users.

## Background

One classic conundrum of microbiology, or actually of biology at large, is the marked borderline that exists between freshwater and marine habitats [1]. Although aquatic environments share many features and ecological parameters, the microbes found throughout both systems have different characteristics at several levels. First, although the major microbial taxa have representation in both, the proportions of each are very different. For instance, the phylum Actinobacteria and the class Betaproteobacteria are two notorious examples of taxa that are more abundant in freshwater [2–4], while classes Alphaproteobacteria and Gammaproteobacteria are more abundant in marine waters [4, 5]. Second, although it might be an artifact of lack of coverage, there are lower-level taxa that appear to be altogether absent in one of the groups of habitats regardless of how abundant they are in the other. Some relevant examples are acI Actinobacteria [6], *Limnohabitans*, and *Polynucleobacter* Betaproteobacteria [7, 8], which dominate freshwater but are absent in marine habitats. LD12 Alphaproteobacteria, including *Ca*. Fonsibacter [9–11], have only been found also in freshwater and estuarine systems, although their relatives in the SAR11 clade are widespread in marine habitats. On the other hand, *Prochlorococcus* species [12] or Gammaproteobacteria groups such as SAR86 [13] are found only in marine ecosystems. The explanation for such differences is not obvious considering the similarity of aquatic pelagic habitats aside from the salinity and the influence of terrestrially derived organic matter [1]. On the other hand, there are reports of multiple marine clades being detected, albeit in small numbers, in freshwater habitats [14–16], and the opposite is true for marine regions neighboring the continents, particularly near large estuaries like the Amazon on the Atlantic coast of Brazil [17] or the Baltic Sea [18, 19]. Thus, the differences cannot be explained by physical isolation. Still, excluding some microbes that can survive and remain rare, such as *Escherichia coli* or *Vibrio cholerae*, there is no known example of microbes of the same species (with > 95% average nucleotide identity, ANI) being found in both types of aquatic environments.

One problem to understand the real differences between these two kinds of aquatic systems is the enormous diversity within each of them. Particularly, freshwater lakes vary in their trophic status (from oligotrophic to highly eutrophic) and other environmental parameters, all of them having profound implications in the taxonomic composition. Recently, we were involved in the first metagenomic study of Lake Baikal, Siberia, Russia [20]. This is the largest and deepest (max. 1600 m, average 1300 m) lake on Earth [21], ultraoligotrophic and with relatively little influence from terrestrial sources (all features that make it similar to marine off-shore waters) while having very low salt content (dominated by Ca2^+^ and HCO_3_^-^, being particularly low in Na^+^ and K^+^) [22–24]. Interestingly, we found some groups with close relatives among bona fide marine lineages, including the first freshwater *Pelagibacter*-like (SAR11 clade) metagenome-assembled genome (MAG) within the typically marine clade I [20]. In previous studies, we compared the pI patterns of this SAR11 MAG [20] and a freshwater *Synechococcus* [25] with their marine closest relatives and, in spite of their relative phylogenetic proximity, noticed significant differences in the global values of their predicted proteome pIs.

The variations in the global proteome pI plots of cells depend on the amino acid overall charge and have important implications on protein structure and properties [26]. It is generally accepted that prokaryotic genomes have a bimodal shape with two maxima [27], one at acidic pH corresponding largely to dissolved proteins (cytoplasmic or secreted) and one at basic pH of the membrane proteins that have a basic (positively charged) domain intracellularly to facilitate the generation of the proton motive force. In between these two peaks, there is a minimum at ca. neutral values that correspond to the intracellular pH at which proteins of equivalent pI value would be the least soluble. In salt-in halophiles, the alkaline peak nearly disappears because they have a large amount of intracellular K^+^ [28]. The adaptation to hypersaline environments (much more saline than seawater) leads to these changes in their inhabitants (halophiles) and has been known for long [29]. Thus, hyperhalophiles such as *Haloquadratum walsbyi* or *Salinibacter ruber* have their proteomes markedly displaced to acidic values [28]. However, marine bacteria and archaea are expected to be salt-out strategists, i.e., that keep most inorganic salts (particularly Na^+^) outside the cell, maintaining a relatively salt-free cytoplasm [28].

The large change in this value detected in the freshwater microbes mentioned above made us wonder if it could be a general phenomenon and what could be the underlying reason for such a broad deviation. There is a current database with pI calculations and amino acid properties for more than > 5000 bacteria and archaea [30], and prior studies identified a correlation between salinity and pIs of microbes [31]. However, there are no studies comparing bona fide freshwater and salt-adapted microbial predicted proteomes including hypothetical proteins derived from metagenomes. Here, filling this gap, we have analyzed in detail some specific cases when closely related microbes by whole genome comparisons have been retrieved from marine and freshwater habitats; furthermore, there is metagenomic evidence (by recruitment of metagenomic reads) showing that they are actually adapted to live in either one or the other environment. We have also dissected these pI values depending on the localization of the proteins and used available three-dimensional models to determine whether there was a preferential location of the charges. Our data confirm that indeed the predicted proteins, regardless of location in the cell, accumulate fewer negative charges in prokaryotes coming from freshwater environments, corresponding to a significant deviation in the amino acidic composition. This fact, among other consequences, implies a large sequence variation that requires long evolutionary times to carry out the transition between marine and freshwater habitats or vice versa.

## Results

### Global pIs of metaproteomes from different aquatic habitats

**Fig. 1:**
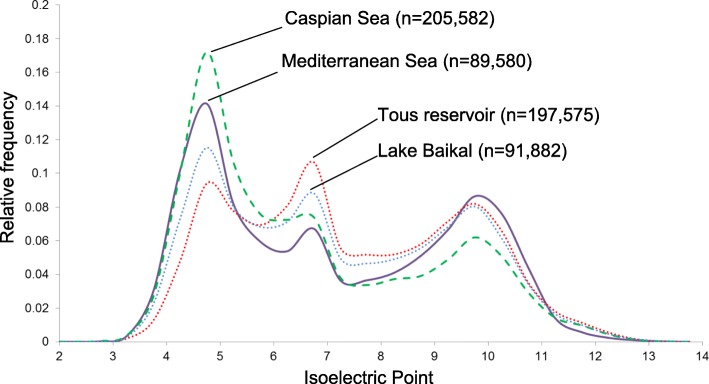
Metaproteome pI versus relative frequency plot of marine (Mediterranean Sea, 30 m deep), brackish (Caspian Sea, 15 m), and freshwater (Tous reservoir, 12–25 m, and Lake Baikal, 20 m) habitats. *N* indicates the number of proteins retrieved from each metaproteome and used in this comparison

A global metagenomic approach was first used to assess if the changes in pI could be detected at the level of the microbial community as a whole. Specifically, we used metagenomic datasets from freshwater (Tous reservoir and Lake Baikal), brackish (Caspian Sea), and marine (Mediterranean Sea) environments from similar depths, which were assembled and annotated, taking all predicted and hypothetical proteins from contigs > 5 kb and obtaining sets of more than 85,000 proteins for each environment (Fig. 1). Interestingly, the three highest peaks were observed at nearly identical pI values (4.5, 6.8, and 9.8) for the different aquatic habitats in spite of the large differences in salinity (ca. 0.05 in freshwater habitats, 1.2 in the Caspian, and 3.8% in the Mediterranean) and community structure [20, 32, 33]. A major difference was observed in the acidic peak, with brackish and marine environments having higher relative frequencies of proteins with lower pIs, compared to their freshwater counterparts. On the other hand, a higher peak of neutral pIs was observable in freshwater systems. Finally, the segment of the plot corresponding to basic pIs (8–9.5 and 11–14) was also higher in freshwaters, although the relative frequencies of pIs from 9.5–11 were a little higher in the Mediterranean Sea. However, these changes could just reflect the variation in the community structure, i.e., very different taxonomic composition depending on the habitat. Therefore, we have analyzed the pI features of the proteomes of related microbes that are bona fide inhabitants of one or the other kind of habitat.

### Overall pI patterns within phyla

**Fig. 2:**
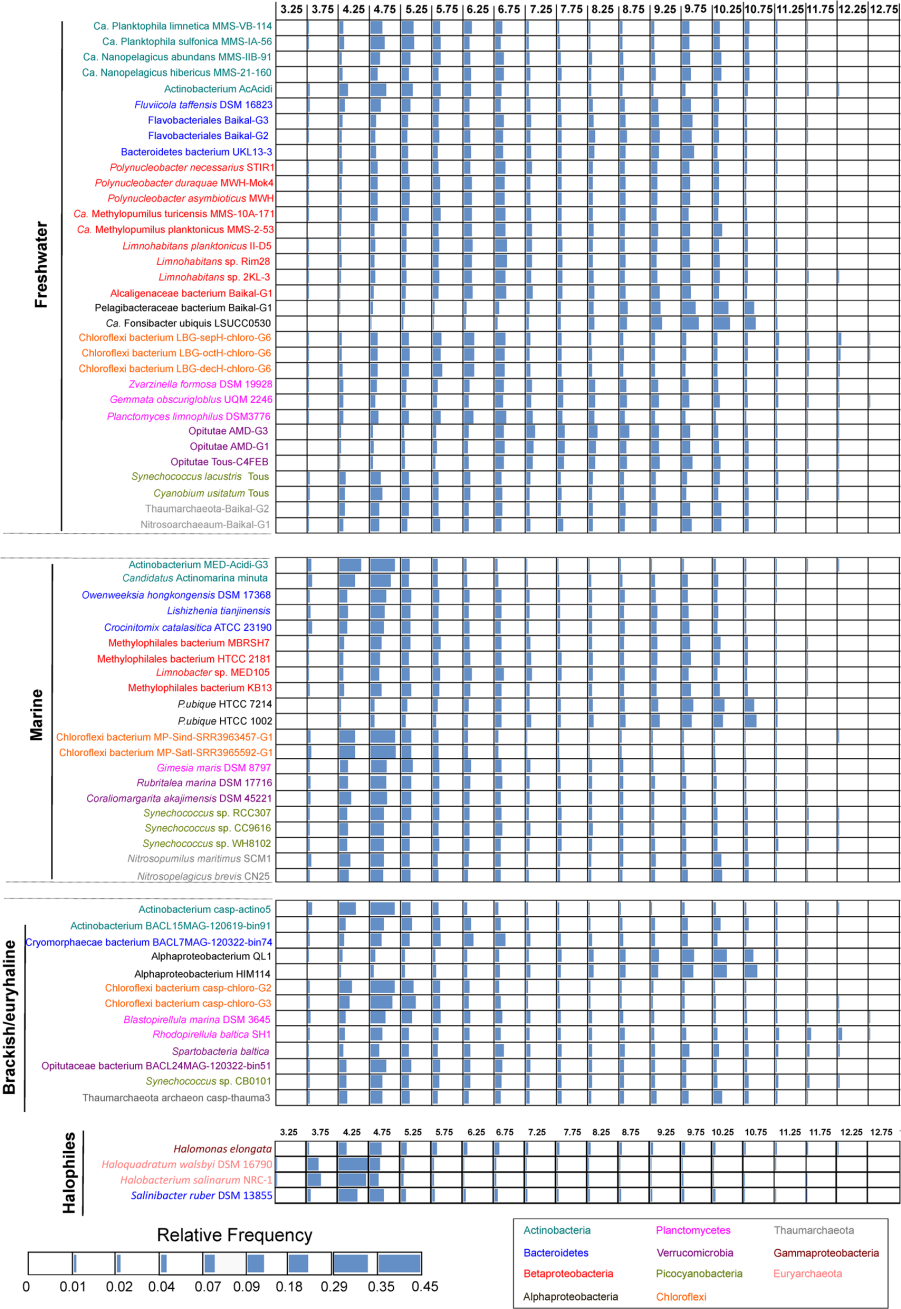
Whole proteome isoelectric point versus relative frequency plot of freshwater, marine, brackish, and halophilic selected prokaryotes. Genomes are color-coded according to their taxonomic affiliation and arranged according to their origin. Bar plots represent the increasing relative frequencies of each isoelectric point value (pI)

To assess if the differences in the global pI distributions were due to the habitat or taxonomic bias, we selected a total of 71 prokaryotes from public databases and compared their overall pI values (Fig. 2). We used examples of bona fide freshwater, brackish, and marine microbes, some of them retrieved as MAGs (> 70% of completeness) from the environments compared in Fig. 1, and others as pure cultures. We selected representatives from class Alphaproteobacteria (SAR11, *Roseobacter*, and Rhodospirillaceae), order Betaproteobacteriales, Chloroflexi, Planctomycetes, Verrucomicrobia, Cyanobacteria (*Synechococcus*/*Cyanobium*), phyla Actinobacteria, Bacteroidetes, and Thaumarchaeota (Fig. 2 and Additional file 1: Figsure S1–S6, see Additional file 2 for extra information on each selected microbe). The Bray-Curtis distances obtained between relative frequencies of pIs and the statistical analysis conducted with PERMANOVA allowed us to compare both habitat and taxonomic distance effects on the relative frequencies of pIs in the dataset of the selected microbes (see the “Methods” section and Additional file 2). We obtained an *R*^2^ of 0.336 and 0.45 for habitat and phyla variables respectively, confirming that there is a taxonomic bias, with an important influence in the pIs, as happens in SAR11 (see Additional file 1: Figure S1 and Additional file 2). However, both variables significantly explained the differences in pI. The principal component analysis (PCA) plot also showed the effect of taxonomic bias (SAR11) and a generally observed different distribution of freshwater and salt-adapted microbes (Additional file 2). In all cases (habitat-specific metaproteomes and phyla by phyla species comparison), we noticed differences between freshwater, brackish, and marine predicted proteome pI patterns (Figs. 1 and 2 and Additional file 1 Figures. S1–S6). Thus, these plots could help to identify in a relatively straightforward manner the salinity of origin of the different microbes without prior knowledge.

First, halophiles present a single acid peak at low pIs (highest among the microbes compared). Second, brackish and marine species tend to show bimodal patterns and display a higher peak of acidic proteins compared to freshwater ones. One exception was SAR11 (Additional file 1: Figure S1), which always presented a higher peak of basic proteins independently of the origin. Third, it is particularly remarkable the high peak of neutral proteins (with pIs ranging from 6 to 8) in some freshwater species, while this peak is very low or absent in salt-adapted species. This was the case of Flavobacteriales, Betaproteobacteriales, Verrucomicrobia, Planctomycetes, or Thaumarchaeota (Additional file 1: Figures. S2, S5, and S6). Our data could facilitate the prediction of a microbe natural ecosystem and it could be established as a rule of thumb to infer the preferred habitat of microbes, particularly useful in mixed systems such as estuaries.

### Pairwise comparisons of close phylogenetic neighbors

**Fig. 3:**
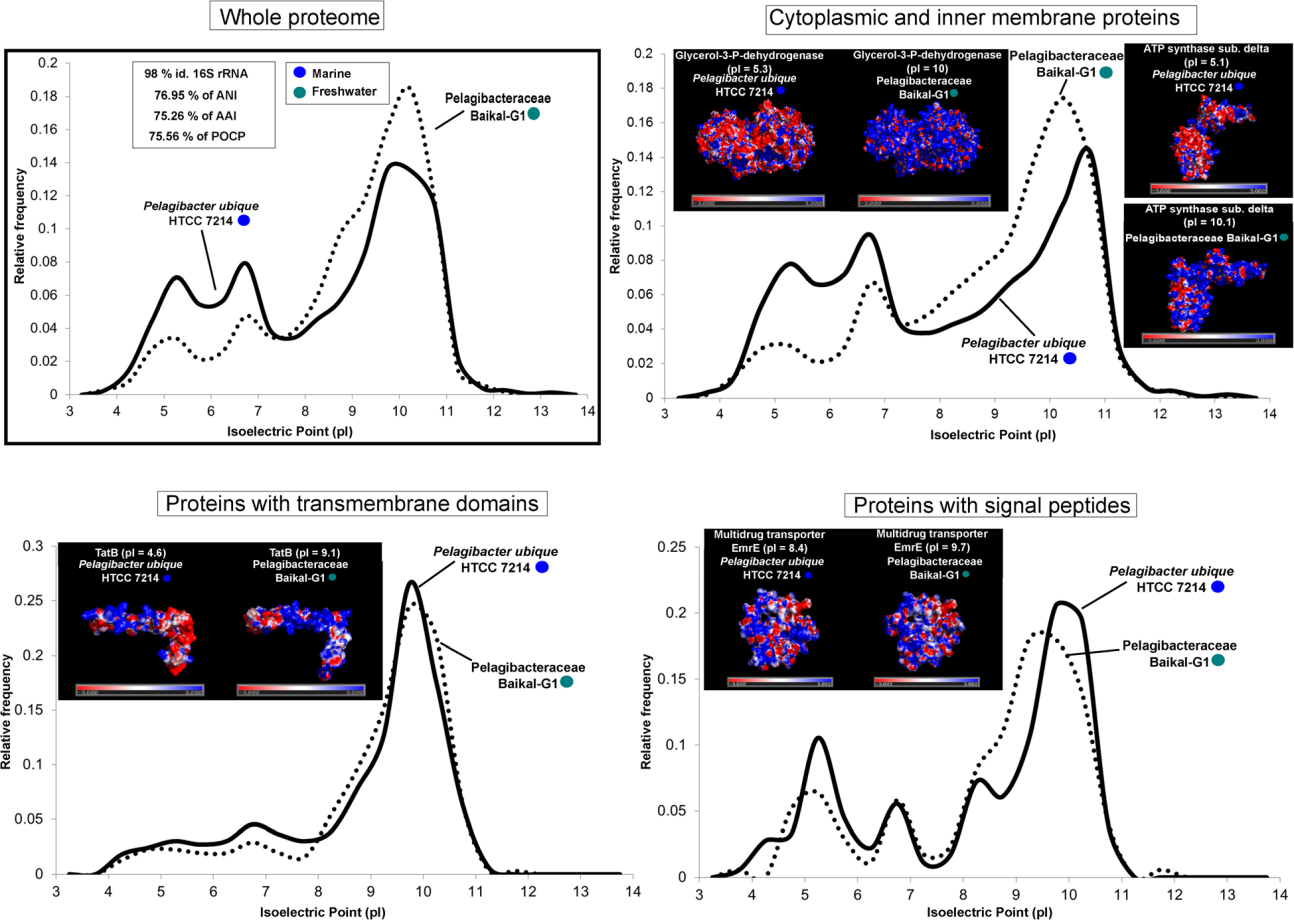
Proteome (whole and at different locations, secreted, cytoplasmic, and transmembrane) pI values versus relative frequency of *P. ubique* HTCC7214 (marine, culture) and Pelagibacteraceae bacterium Baikal-G1 (freshwater, MAG). Insets show tridimensional electrostatic surface potential 3D models of individual proteins selected for each location (secreted, cytoplasmic, and transmembrane). The potentials were colored from − 3 kcal mol^−1^(red) to + 3 kcal mol^−1^ (blue). Values of ANI, AAI, POCP, and percentage of identity of 16S rRNA for each couple of microbes are also indicated

**Fig. 4:**
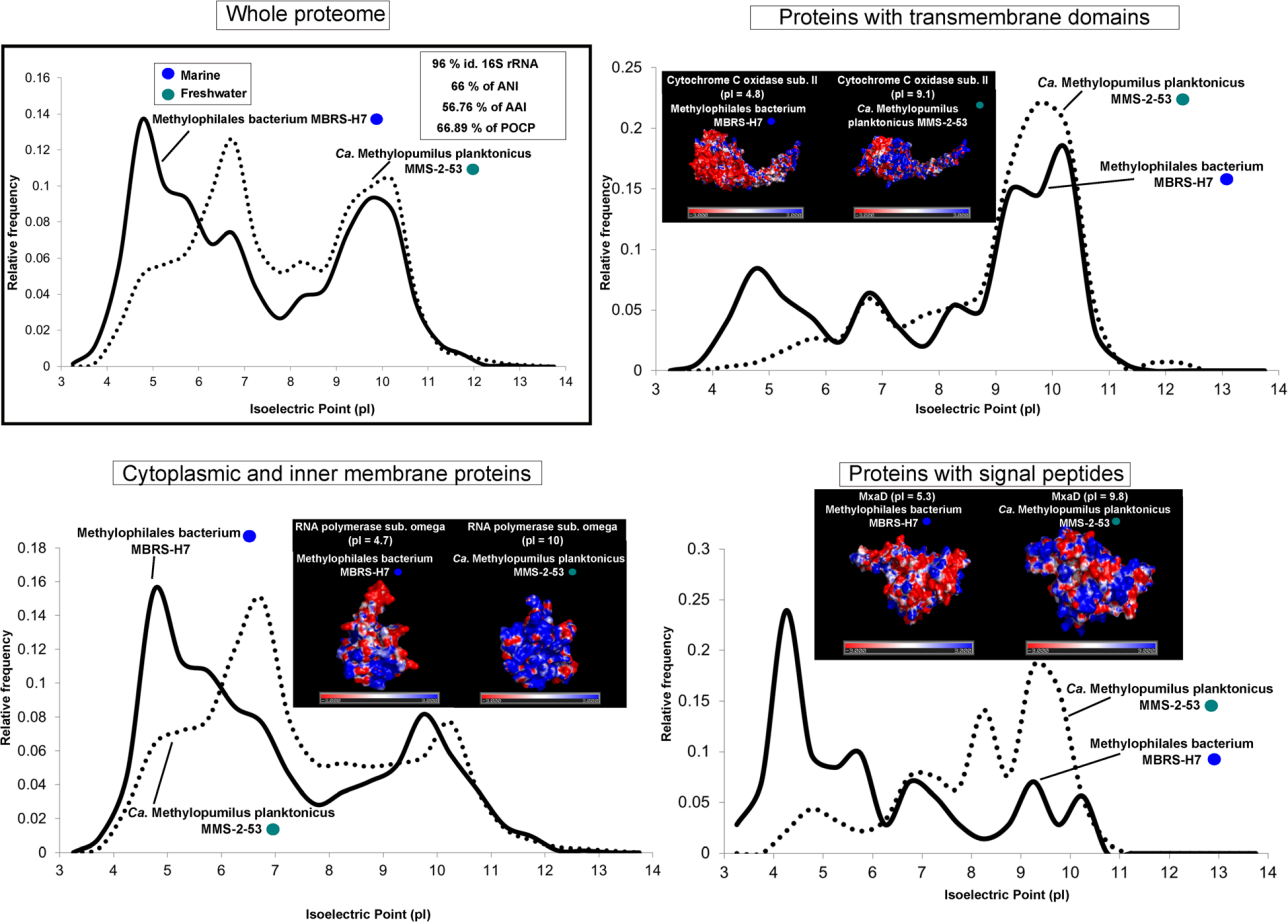
Proteome (whole and at different locations, secreted, cytoplasmic, and transmembrane) pI values versus relative frequency of *Ca*. Methylopumilus planktonicus MMS-2-53 (freshwater, culture) and Methylophilales bacterium MBRS-H7 (marine, culture). Insets show tridimensional electrostatic surface potential 3D models of individual proteins selected for each location. The potentials were colored from − 3 kcal mol^−1^(red) to + 3 kcal mol^−1^ (blue). Values of ANI, AAI, POCP, and percentage of identity of 16S rRNA for each couple of microbes are also indicated

**Fig. 5:**
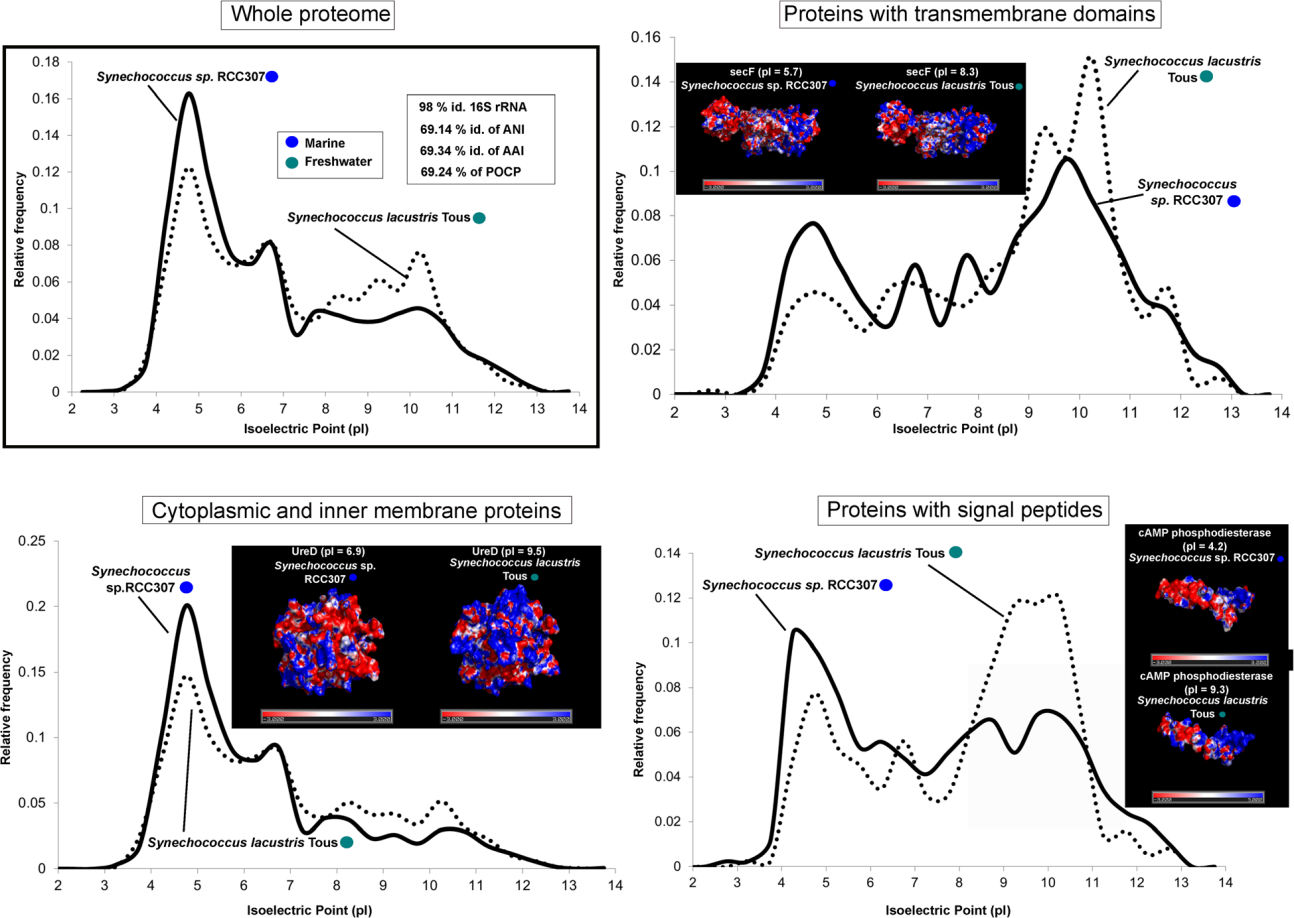
Proteome (whole and at different locations, secreted, cytoplasmic, and transmembrane) pI values versus relative frequency of *Synechococcus lacustris* Tous (freshwater, culture) and *Synechococcus* sp. RCC307 (marine, culture). Insets show tridimensional electrostatic surface potential 3D models of individual proteins selected for each location. The potentials were colored from − 3 kcal mol^−1^ (red) to + 3 kcal mol^−1^ (blue). Values of ANI, AAI, POCP, and percentage of identity of 16S rRNA for each couple of microbes are also indicated

**Fig. 6:**
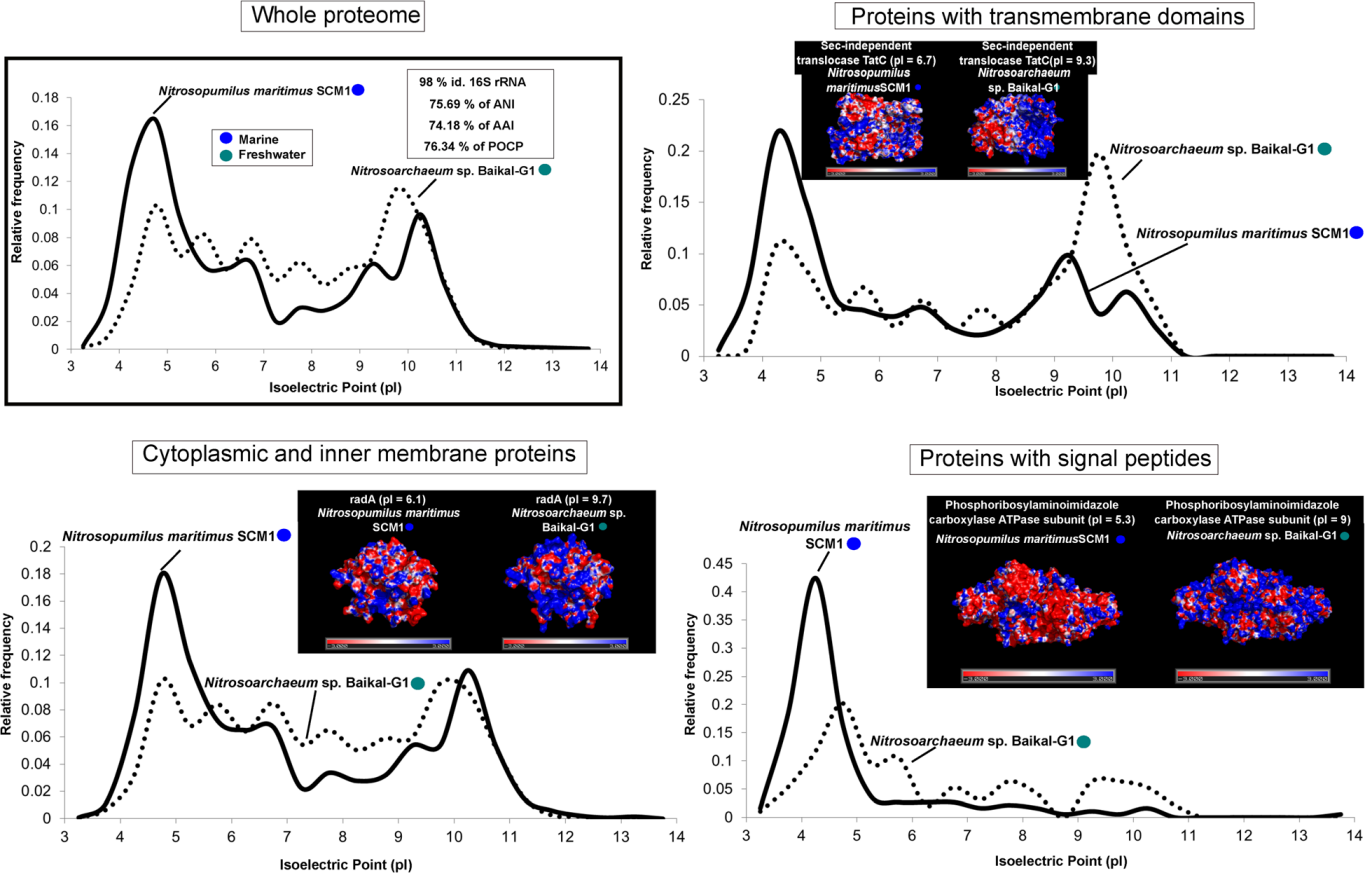
Proteome (whole and at different locations, secreted, cytoplasmic, and transmembrane) pI values versus relative frequency of *Nitrosopumilus maritimus* SCM1 (marine, culture) and *Nitrosoarchaeum* sp. Baikal-G1 (freshwater, MAG). Insets show tridimensional electrostatic surface potential 3D models of individual proteins selected for each location (secreted, cytoplasmic, and transmembrane). The potentials were colored from − 3 kcal mol^−1^(red) to + 3 kcal mol^−1^ (blue). Values of ANI, AAI, POCP, and percentage of identity of 16S rRNA for each couple of microbes are also indicated

We selected pairs of microbes that are as closely associated phylogenetic neighbors as available (same family or genera, whenever possible), but one of them is freshwater and the other marine inhabitants. In these cases, the effect of the taxonomic distance was reduced to the minimum presently available in databases. Thus, we could compare two species from the family Nitrosopumilaceae (*Nitrosoarchaeum* sp. Baikal-G1, MAG, vs *Nitrosopumilus maritimus* SCM1, culture), two SAR11 members from family Pelagibacteraceae (Pelagibacteraceae bacterium Baikal-G1, MAG, vs *Pelagibacter ubique* HTCC7214, culture), two picocyanobacteria from the order Synechococcales (*Synechococcus* sp. RCC307, culture, vs *Synechococcus lacustris* Tous, culture), and finally two species from the family Methylophylaceae (Methylophilales bacterium MBRS-H7, culture vs *Methylopumilus planktonicus* MMS-2-53, culture). We chose pairs of microbes with similar cell and genome sizes displaying similar metabolic and ecological roles in the environment to reduce to the minimum other factors. Values of average nucleotide identity (ANI), average amino acid identity (AAI), 16S rRNA gene identity, and percentage of conserved proteins (POCP) were also calculated for each pair. We divided the proteome into three categories: cytoplasmic and inner membrane proteins that are submitted to the cytoplasmic environment, proteins with transmembrane domains, and secreted (with signal peptide), i.e., exposed to the extracellular environment. The average pIs were also calculated for these three categories. The differences between freshwater and marine microbes appear clear at all levels (Figs. 3, 4, 5, and 6). We found in these pairs of microbes whose ANI varied between 66 and 77% that the AAI values were similar (when not lower) than the nucleotide identity. This is contrary to the expectations in comparisons of phylogenetic neighbors that tend to have more similarity at the level of amino acids than nucleotides [34, 35]. This also indicated a major shift in the composition of amino acids of the core genome (shared genes, see below).

### Changes in the amino acid composition

The variations in the amino acid composition (expressed as Mole% for each microbe) in the compared pairs are noteworthy (Additional file 1: Figure S7). A general trend that is conserved in the four cases is the higher percentage of acid amino acids (aspartic and glutamic) in the marine representatives (from 0.6 to 1.4% higher). This is in agreement with the overall higher peak of acidic proteome pIs in these salt-adapted microbes. Actually, the global percentage of charged amino acids comprising both acid and basic types (aspartic and glutamic acids, lysine, histidine, and arginine) is higher in marine microbes, i.e., the acid increase in marine is more accentuated than the basic amino acids in freshwater representatives. However, three out of four freshwater microbes analyzed here display higher percentages of basic amino acids compared with their marine relatives. The only exception observed is the case of *S. lacustris* Tous, which presents a nearly identical percentage of basic amino acids when compared with its marine relative *Synechococcus* RCC307. Nevertheless, there are noticeable differences, for instance, *S. lacustris* shows a higher percentage of lysine (K) residues on its whole proteome, while RCC307 compensates this decrease in lysine by having more arginine (R) residues, resulting in practically the same percentage of total basic amino acids in both genomes. On the other hand, the higher percentage of basic residues in Methylophilales, Pelagibacteraceae, and archaeal Nitrosopumilaceae genomes is significant. *Nitrosoarchaeum* sp. Baikal-G1 presents a higher percentage of all three basic amino acids, compared to its marine relative *Nitrosopumilus maritimus* SCM1. *Ca*. Methylopumilus planktonicus MMS-2-53 displays a higher percentage of arginine and histidine, but slightly lower lysine compared to its marine relative. Hence, a global picture contemplating these variations in the overall pI plots, electrostatic surface potential of proteins, and percentages of basic/acid amino acids could help predicting the freshwater or salt-adapted origin of a novel microbe of unknown source.

### Changes are located at the surface of proteins

In salt-in halophiles, the changes in aminoacidic composition are concentrated at the level of the protein surface producing an even sharper change in the electrostatic surface potential [36]. Therefore, we analyzed the case of some individual protein homologs for which there were tridimensional models to assess if the difference in the predicted global pI was concentrated at the level of the protein surface. We chose homologs with a marked difference in the global pI between freshwater and marine representatives, and only proteins with well-established tertiary structures (retrieved from SWISS-MODEL) were considered. Indeed, we observed a higher accumulation of negative charges (acid amino acids) on the surface of proteins from marine microbes, while positive electrostatic potentials (with more basic amino acids) are seen in freshwater microbes (Figs. 3, 4, 5, and 6). We are showing only some individual protein examples for each category/prokaryote; however, many other protein homologs presented substantial differences in their pIs (see Additional file 3). Thus, it appears that the electrostatic surface potential between pairs of homologs differs significantly between marine and freshwater species. These differences are even more evident when introducing halophiles into the comparison (Additional file 1: Figure S8). These significant variations were apparent in all three categories: cytoplasmic, membrane, and secreted proteins. However, the differences in electrostatic surface potential were more marked in the secreted > cytoplasmic > membrane, which indicates a more radical change in the extracellular than in the intracellular environment between freshwater and marine microbes.

### Pan-genome pI distribution

We also calculated the pIs for each category (core and flexible proteins) to assess if the change in the global proteome might be due to variations affecting homologous proteins as those shown above or it could be an extreme consequence of a change in the differential gene pool present in freshwater or marine microbes, i.e., it could be due to the flexible or core genes [37, 38]. Therefore, we analyzed the global pI plots of both components in the four close relative comparisons (Additional file 1: Figure S9). Indeed, marine representatives always had a higher peak of acidic proteins, independently if the coding genes belong to the core or flexible genome component. Actually, only the case of *Ca*. Methylopumilus displayed a different pattern between core and flexible genes (both being more displaced towards neutrality-basicity compared to their marine homolog). In the other cases, there were no differences altering significantly the patterns observed in whole genomes. The flexible genome had in three of the examples a higher basic peak, probably reflecting enrichment in membrane proteins, largely transporters, and sensors that are typical components of the flexible genome (involved in habitat-adaptation and niche-occupancy) [39].

## Discussion

Previous studies showed that bacteria and archaea, independently of their origin, presented a bimodal pI pattern, while eukaryotes showed a trimodal pattern [27]. Others have shown a multi-modal distribution picture of the pIs in different organisms as a consequence of the chemical properties of the different amino acids (rather than sequence evolution) [26] or as a result of discrete pKr values for the amino acids [40]. Our observations indicate that the pI pattern varies significantly among microbes, having cases of unimodal (salt-in halophiles) and bimodal patterns (most of the microbes analyzed here) to multimodal (Thaumachaeaota, Verrucomicrobia, Planctomycetes, or Betaproteobacteriales).

The pI of a certain protein is a major indicator of the properties of the macromolecule. It determines the water solubility of the protein as well as the interactions with the chemical environment. Typically, intracellular pH of microbes (including alkaliphilic or acidophilic ones) is near neutrality [41, 42], and proteins are less soluble at pH values near their pI. Thus, cytoplasmic and secreted proteins, that mostly work in a soluble form, tend to have pIs far from neutrality, mostly acidic [41]. On the other hand, membrane proteins only interact with water in their exposed domains and tend to have alkaline pIs to compensate for the positive charge outside of the membrane created by the proton gradient [43]. The consistent difference that we have detected between freshwater and marine microbes indicates a significant change in one of these two aspects of cell biology, either the intracellular pH or the bioenergetics of the cell (perhaps both). Another factor that likely interacts with the protein charge is the presence of other solutes in the water phase at both sides of the membrane. Most cells maintain a significant concentration of K^+^ cations inside while keeping Na^+^ outside. Typically, marine microbes would need higher intracellular potassium concentrations in order to compensate the sodium ions abundant in the extracellular environment. Depending on the cation concentration, soluble proteins need to have more or less negative charges to maintain a proper hydration sphere [44]. This is why halophiles with salt-in strategies must have very acidic soluble proteins [28]. Freshwater must impose limitations to the accessibility to the main cellular cations, particularly Na^+^, that might be limiting in salt-poor environments like Lake Baikal [22]. These conditions could lead to adaptations consisting of less intracellular potassium. It is thus not surprising that a less acidic proteome might be favorable for freshwater microbes. Other predicted physiological differences between the two types of aquatic microbes include the preference for H^+^- over Na^+^-based electron or nutrient transport mechanisms [41], but this is unlikely to have effects over the global proteome as described here.

The kind of analysis that we have done in this work has previously been hampered by the lack of close relatives specialized in living in either freshwater or marine habitats. However, provided that most of the microbial examples that we have used are difficult to retrieve in pure culture and are only available as genomes, it is not feasible to carry out physiological or biophysical experiments that could clarify the meaning of the patterns that we have found. Hence, our hypotheses rely on predicted proteins from either pure cultures or MAGs. For instance, one crucial point would be determining the intracellular K^+^ concentration, which to date has been only done in *E. coli* [45], a marine *Pseudomonas* [46], and some halophiles [47]. Similarly, we need to understand how different microbes regulate their cytoplasmic pH in response to environmental changes. However, there is a significant difficulty in measuring the cytoplasmic pH of microbes under growth conditions [42]. Furthermore, some microbes undergo small variations in the pH of their cytoplasm (up to 0.1 units per pH unit change), while others such as *E. coli* or *Coxiella burnetti* are subjected to much wider changes [48, 49].

As could be expected, there is a taxonomic component in the pI patterns, for example, SAR11 clade members tend to have the pI plot displaced towards basic values (Additional file 1: Figure S1, see Additional file 2). That streamlined bacteria, independently of their origin, should have a tendency to basicity in their pIs is to be expected (Additional file 1: Figure S10) considering their higher surface/volume ratio, which leads to a higher membrane/cytoplasmic proteins ratio. Still, even in these cases, the differential value in the freshwater-marine comparison was detectable (i.e., regardless of the pI range always marine have more acidic average values). This general pattern was confirmed by the amino acid composition that shows common trends in organisms as phylogenetically distant as *Pelagibacter* and Thaumarchaeota. It was also remarkable that in closely related microbes but from different origin (marine or freshwater), AAI was similar and (in most cases) lower than ANI, i.e., amino acid similarity is lower than nucleotide identity. This was also observed in freshwater, euryhaline, and marine *Synechococcus*/*Cyanobium* genera [50]. This is the opposite of what we found when comparing similarly distant microbes but living in the same type of aquatic habitats, such as freshwater acI Actinobacteria [6]. The values are consistently AAI < ANI as could be expected from the existence of neutral changes due to the degeneration of the genetic code.

Our work underscores the important changes that a microbe must suffer to get adapted to freshwater from a marine habitat or vice versa. If many (or most) proteins change in their amino acidic composition, the number of changes, i.e., the evolutionary time involved, have to be large. Although several studies assured that marine-freshwater transitions tended to be infrequent [51, 52], it has been proven that some close relatives to marine microbes are found in freshwater habitats (SAR11 Pelagibacteraceae, Rhodobacteraceae, and Flavobacteria) [14–16, 20]. Furthermore, microbial transitions have also been recorded in brackish ecosystems such as Baltic Sea [18], in which some species of brackish origin transit to either marine or freshwater systems. Therefore, the transition, although demanding, could have happened at some stages in the long evolutionary history of microbes, but the adaptation to be freshwater or marine adapted is a crucial evolutionary decision that every species take.

## Conclusions

There is a large change in amino acid composition among microbes depending on whether they live in marine or freshwater habitats. The change can already be detected by relatively low values of AAI (compared to ANI) and is reflected by a major shift in the pI pattern of the cell predicted proteome, with an increase in the acidic peak in the marine microbes and another (albeit more moderate) in the neutral and basic peaks for the freshwater ones. These changes occur also in closely related microbes, i.e., they do not reflect a taxonomic bias. Furthermore, we have been able to see changes in individual proteins with 3D models and their overall surface electrostatic potential, indicating that the changes tend to accumulate on the surface of the protein, particularly when they are soluble (cytoplasmic or secreted).

We propose that our results indicate an important change in cell physiology due to the absence of salts in the freshwater habitats. This absence might imply specific requirements of membrane characteristics (membranes could change in composition when exposed to the absence of salts in significant amounts since the stability of lipid bilayers could be affected), bioenergetics (differences in the electrochemical gradient across the membrane), intracellular pH (a change in the intracellular pH would modify the solubility of the proteins), or K^+^ concentration (requiring less acidity to compensate the positive charge of intracellular cations) or a combination of these or other components of cell biology which apply throughout the prokaryotic domain, bacteria, and archaea.

## Methods

### Metagenomic datasets and bacterial genomes used in this work

All metagenomic datasets used in this work are publicly available in the NCBI/SRA databases: Mediterranean Sea [33], Caspian Sea [32], Lake Baikal [20], Tous reservoir [25]. All bacterial and archaeal genomes used in this study, together with their accession/Genbank number (NCBI), habitat, isolation/origin, reference, type of genome, and phylum are shown in the Additional file 2. The eight genomes used in the protein-by protein-based comparison were previously published: *Synechococcus lacustris* Tous [50], *Synechococcus* sp. RCC307 [53], *Methylopumilus planktonicus* MMS-2-53 [54], *Methylophilales bacterium* MBRSH7 [55], Pelagibacteraceae bacterium Baikal-G1 [20], *Pelagibacter ubique* HTCC 7214 (ASM70138v1), *Nitrosopumilus maritimus* SCM1 [56], and *Nitrosoarchaeum* sp. Baikal-G1 [20].

### Protein isoelectric point determination

The isoelectric point calculations and amino acid features of each predicted protein and microbe were calculated with the software Pepstats from the EMBOSS package [57]. To determine the pI distribution from metaproteomes, we obtained all proteins from the assembled contigs larger than 5 kb, which were representative of the different taxa inhabiting each ecosystem [20, 32, 33]. We used at least 85,000 proteins per metagenome (Mediterranean Sea 30 m, Lake Baikal 20 m, Caspian Sea 15 m, and Tous reservoir 12–25 m).

### Statistical analysis of the different habitat adapted microbes

Bray-Curtis distances between the relative frequencies of the pIs were calculated to evaluate if the differences in pIs between the 71 habitat-adapted microbes (Fig. 2) were due to the habitats (freshwater, marine, brackish, halophile) or taxonomic classification (phyla). Then, we performed a normalization of the dataset and a PERMANOVA analysis with Vegan R-package [58]. We also obtained a two-dimensional principal component analysis plot with FactoMineR package [59] and calculated average and standard deviation values between all relative frequencies of different microbes for acid (3.25–6.25), neutral (6.75–7.25), and basic (7.75–13.75) pIs. All the statistical analyses are shown in Additional file 2.

### Category classification of different proteins

Each protein was categorized into cytoplasmic/inner membrane, proteins with transmembrane domains, and proteins with signal peptides according to the SignalP [60] and Phobius [61] tools predictions. The pIs of the different proteins, their transmembrane domain topology, and presence/absence of signal peptides for the eight microbes used in this comparison are shown in Additional file 3.

### Structure homology modeling and determination of the electrostatic surface potential of different proteins

The selected proteins for the pair-wise microbe comparison were first modeled for their tertiary structure with the SWISS-MODEL online tool [62–64]. The extracted PDB was then visualized with PYMOL [65], and the electrostatic surface potential was calculated with APBS tool [66]. The surface potentials were mapped from − 3 kcal mol^−1^ (red) to + 3 kcal mol^−1^ (blue).

### Pan-genome analysis

The different freshwater and marine genomes used in the structural comparison were also subjected to a pan-genome analysis. Core and flexible genomes were determined with OrthoMCL and getHomologues software [67, 68].

## Additional files

Additional file 1: **Figure S1.** Whole proteome pI versus relative frequency plot of some representatives from different habitats of the class Alphaproteobacteria. A) Rhodospirillaceae and *Roseobacter* clades and B) SAR11 clade. **Figure S2.** Whole proteome pI versus relative frequency plot of some representatives from different habitats of A) phylum Bacteroidetes and B) Order Betaproteobacterales. **Figure S3.** Whole proteome pI versus relative frequency plot of some representatives from different habitats of A) phylum Chloroflexi and B) phylum Actinobacteria. **Figure S4.** Whole proteome pI versus relative frequency plot of some representatives from different habitats of A) Genera Synechococcus/Cyanobium and B) Assorted halophiles (bacteria and archaea). **Figure S5.** Whole proteome pI versus relative frequency plot of some representatives from different habitats of A) phylum Verrucomicrobia and B) phylum Planctomycetes. **Figure S6.** Whole proteome pI versus relative frequency plot of some representatives from different habitats of the phylum Thaumarchaeota. **Figure S7.** Star diagrams and amino acid composition of prokaryotic relatives from marine and freshwater origin. **Figure S8.** Structural model of proteins from different habitat-adapted archaea. Insets show electrostatic surface potential 3D models of N5-carboxyaminoimidazole ribonucleotide synthetase (secreted protein) and radA (cytoplasmic). The potentials were colored from -3 kcal mol-1(red) to +3 kcal mol-1 (blue). Whole proteome pI versus relative frequency plot of *Haloquadratum walsbyi* DSM 16790 (halophile, culture), *Nitrosopumilus maritimus* SCM1 (marine, culture), *Nitrosoarchaeum* sp. Baikal-G1 (freshwater, MAG). **Figure S9.** Isoelectric point versus relative frequency plot of the pan-genome (core and flexible genome) of freshwater and marine prokaryotes. N indicates the number of proteins in either core or flexible genomes. A) *P.ubique* HTCC 7214 and Pelagibacteraceae bacterium Baikal-G1. B) *Ca*. Methylopumilus planktonicus MMS-2-53 and Methylophilales bacterium MBRS-H7. C) *Synechococcus* sp. RCC307 and *Synechococcus lacustris* Tous. D) *Nitrosopumilus maritimus* SCM1 and *Nitrosoarchaeum* sp. Baikal-G1. **Figure S10.** Whole proteome pI versus relative frequency plot of some streamlined bacteria from different habitats. (PDF 4739 kb)

Additional file 2: Prokaryotic genomes used in this study. Name of the genome, accession/Genbank number (NCBI), habitat, isolation/origin, reference, type of genome and taxonomy fields are provided. Habitat, phyla and Isoelectric point relative frequencies of each microbe. Statistical analysis of the compared dataset (Bray-Curtis+PERMANOVA, Principal Component Analysis, Average + Standard Deviation). (XLSX 60 kb)

Additional file 3: Classification and pI values of the different types of proteins retrieved from the eight prokaryotes used in the electrostatic surface potential and pI comparisons. The pIs of the different proteins, their transmembrane domain topology and presence/absence of signal peptides are shown. (XLSX 784 kb)

## Data Availability

All metagenomic datasets and microbial genomes used in this work are publicly available in the NCBI/SRA databases.

## Abbreviations

AAI: Average amino acid identity
ANI: Average nucleotide identity
MAG: Metagenome-assembled genome
pI: Isoelectric point
POCP: Percentage of conserved proteins
