# Supplementary material for: Marine-freshwater prokaryotic transitions require extensive changes in the predicted proteome
Source: Microbiome. 2019 Aug 22;7:117. doi: 10.1186/s40168-019-0731-5 (PMC6706942; doi:10.1186/s40168-019-0731-5)
Supplement: Supplementary file 1 — Figure S1. Whole proteome pI versus relative frequency plot of some representatives from different habitats of the class Alphaproteobacteria. A) Rhodospirillaceae and Roseobacter clades and B) SAR11 clade. Figure S2. Whole proteome pI versus relative frequency plot of some representatives from different habitats of A) phylum Bacteroidetes and B) Order Betaproteobacterales. Figure S3. Whole proteome pI versus relative frequency plot of some representatives from different habitats of A) phylum Chloroflexi and B) phylum Actinobacteria. Figure S4. Whole proteome pI versus relative frequency plot of some representatives from different habitats of A) Genera Synechococcus/Cyanobium and B) Assorted halophiles (bacteria and archaea). Figure S5. Whole proteome pI versus relative frequency plot of some representatives from different habitats of A) phylum Verrucomicrobia and B) phylum Planctomycetes. Figure S6. Whole proteome pI versus relative frequency plot of some representatives from different habitats of the phylum Thaumarchaeota. Figure S7. Star diagrams and amino acid composition of prokaryotic relatives from marine and freshwater origin. Figure S8. Structural model of proteins from different habitat-adapted archaea. Insets show electrostatic surface potential 3D models of N5-carboxyaminoimidazole ribonucleotide synthetase (secreted protein) and radA (cytoplasmic). The potentials were colored from -3 kcal mol-1(red) to +3 kcal mol-1 (blue). Whole proteome pI versus relative frequency plot of Haloquadratum walsbyi DSM 16790 (halophile, culture), Nitrosopumilus maritimus SCM1 (marine, culture), Nitrosoarchaeum sp. Baikal-G1 (freshwater, MAG). Figure S9. Isoelectric point versus relative frequency plot of the pan-genome (core and flexible genome) of freshwater and marine prokaryotes. N indicates the number of proteins in either core or flexible genomes. A) P.ubique HTCC 7214 and Pelagibacteraceae bacterium Baikal-G1. B) Ca. Methylopumilus planktonicus MMS-2-53 and Methyloph [file 40168_2019_731_MOESM1_ESM.pdf]

Additional file 1

Marine-freshwater prokaryotic transitions require extensive changes in the predicted proteome

Pedro J. Cabello-Yeves[1] and Francisco Rodriguez-Valera[1*]

[1] Evolutionary Genomics Group, Departamento de Producción Vegetal y Microbiología, Universidad Miguel Hernández, San Juan de Alicante, 03550 Alicante, Spain.

*Correspondence: Francisco Rodríguez-Valera, E-mail: frvalera@umh.es

Phone +34-965919313, Fax +34-965 919457

Keywords: Isoelectric point, marine-freshwater transition, metaproteome, acid shift, electrostatic surface potential.

Running title: Marine-freshwater transitions predicted proteome

This PDF file includes: Figs. S1 to S10.

Other Additional files for this manuscript include the following:
Additional files 2 and 3

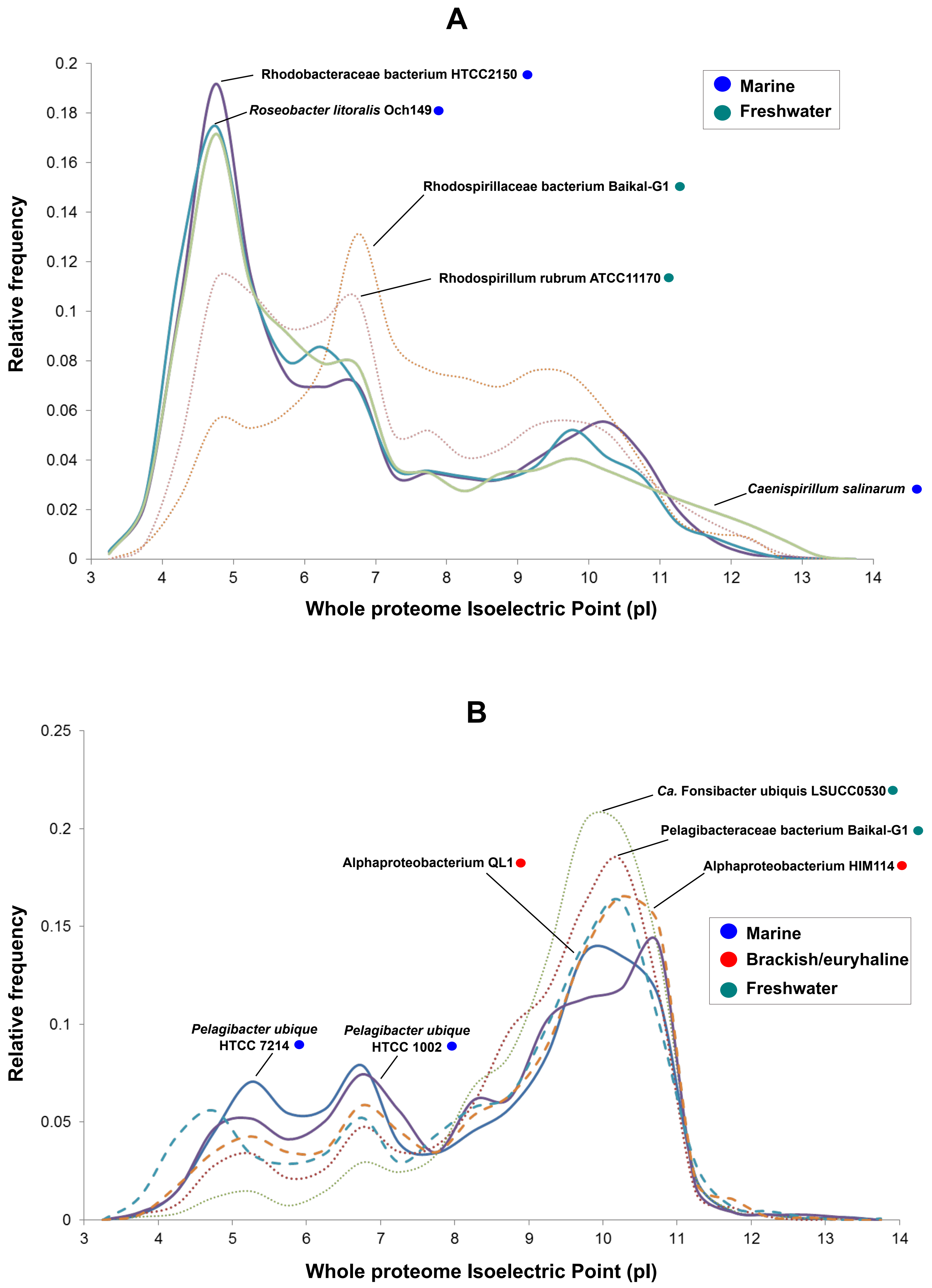

Figure S1. Whole proteome pI versus relative frequency plot of some representatives from different habitats of the class Alphaproteobacteria. A) Rhodospirillaceae and *Roseobacter* clades and B) SAR11 clade.

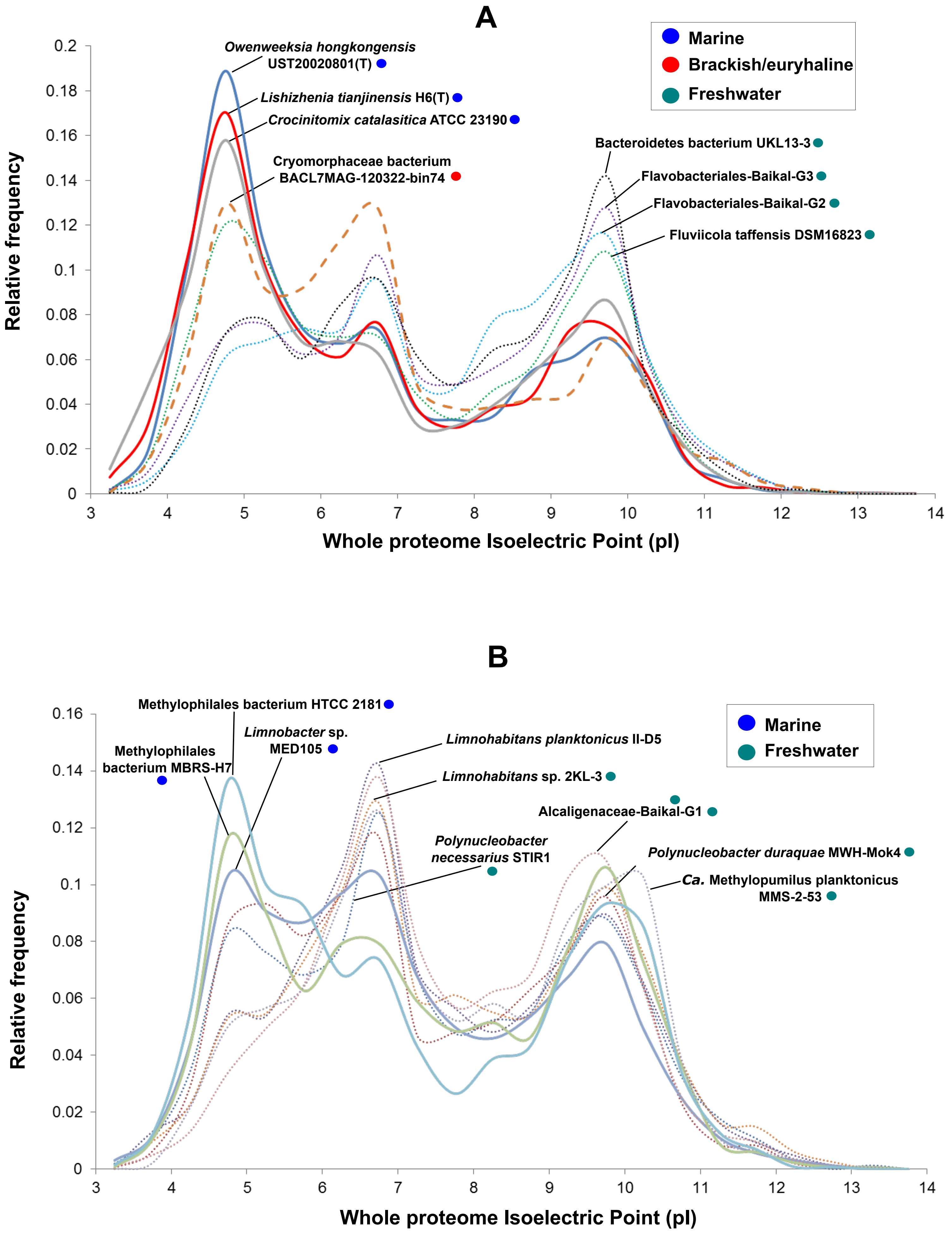

Figure S2. Whole proteome pI versus relative frequency plot of some representatives from different habitats of A) phylum Bacteroidetes and B) Order Betaproteobacterales.

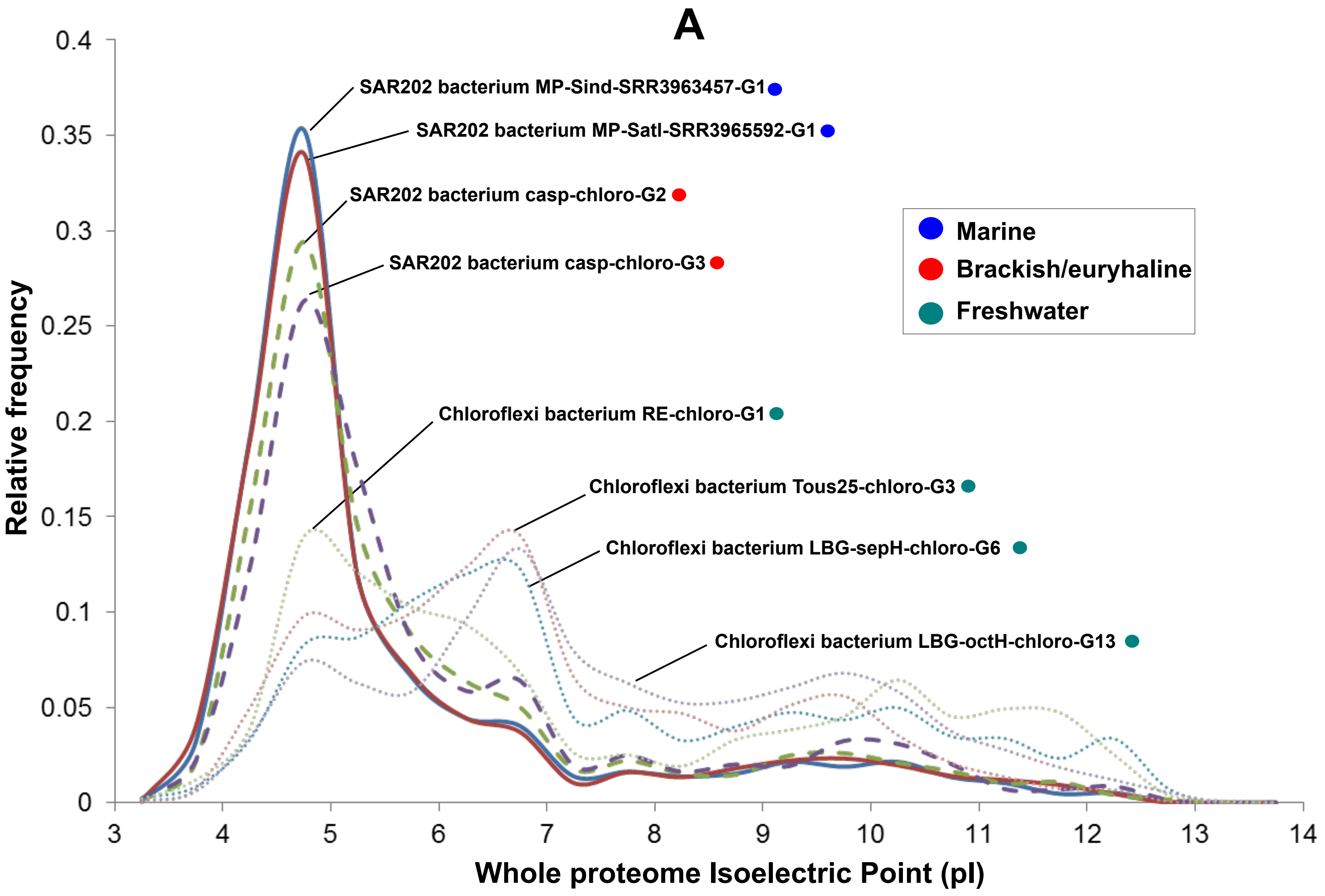

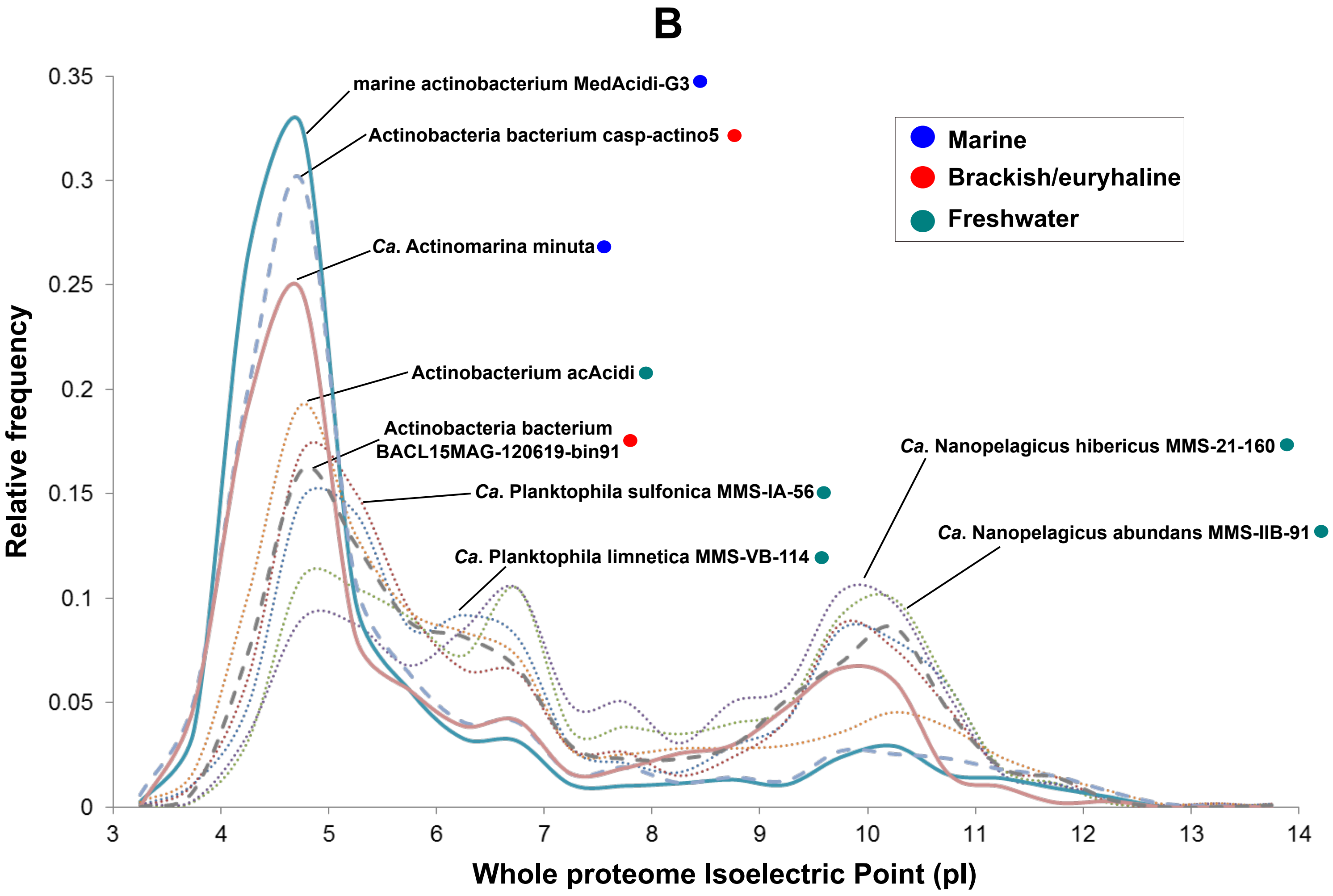

Figure S3. Whole proteome pI versus relative frequency plot of some representatives from different habitats of A) phylum Chloroflexi and B) phylum Actinobacteria.

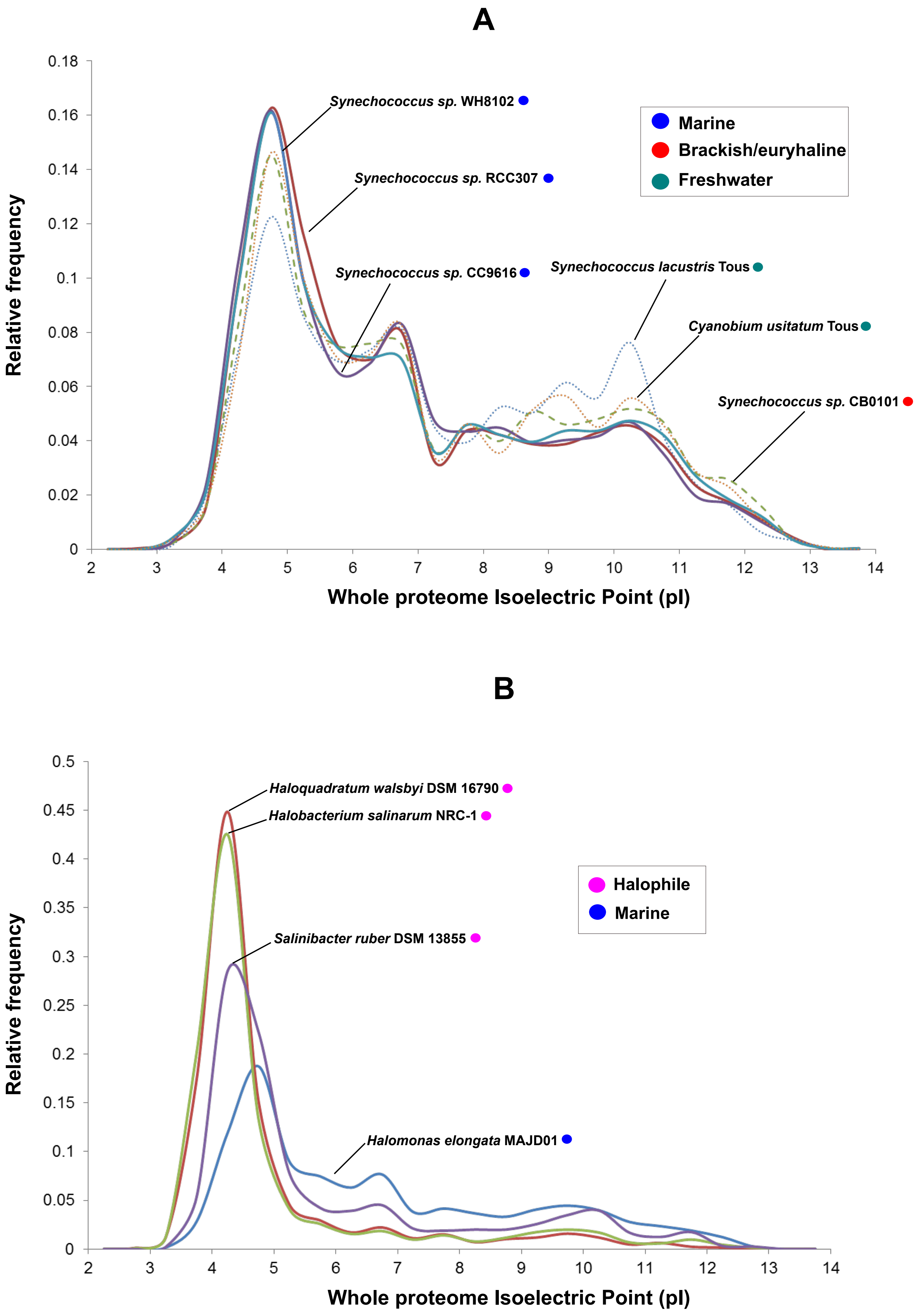

Figure S4. Whole proteome pI versus relative frequency plot of some representatives from different habitats of A) Genera Synechococcus/Cyanobium and B) Assorted halophiles (bacteria and archaea).

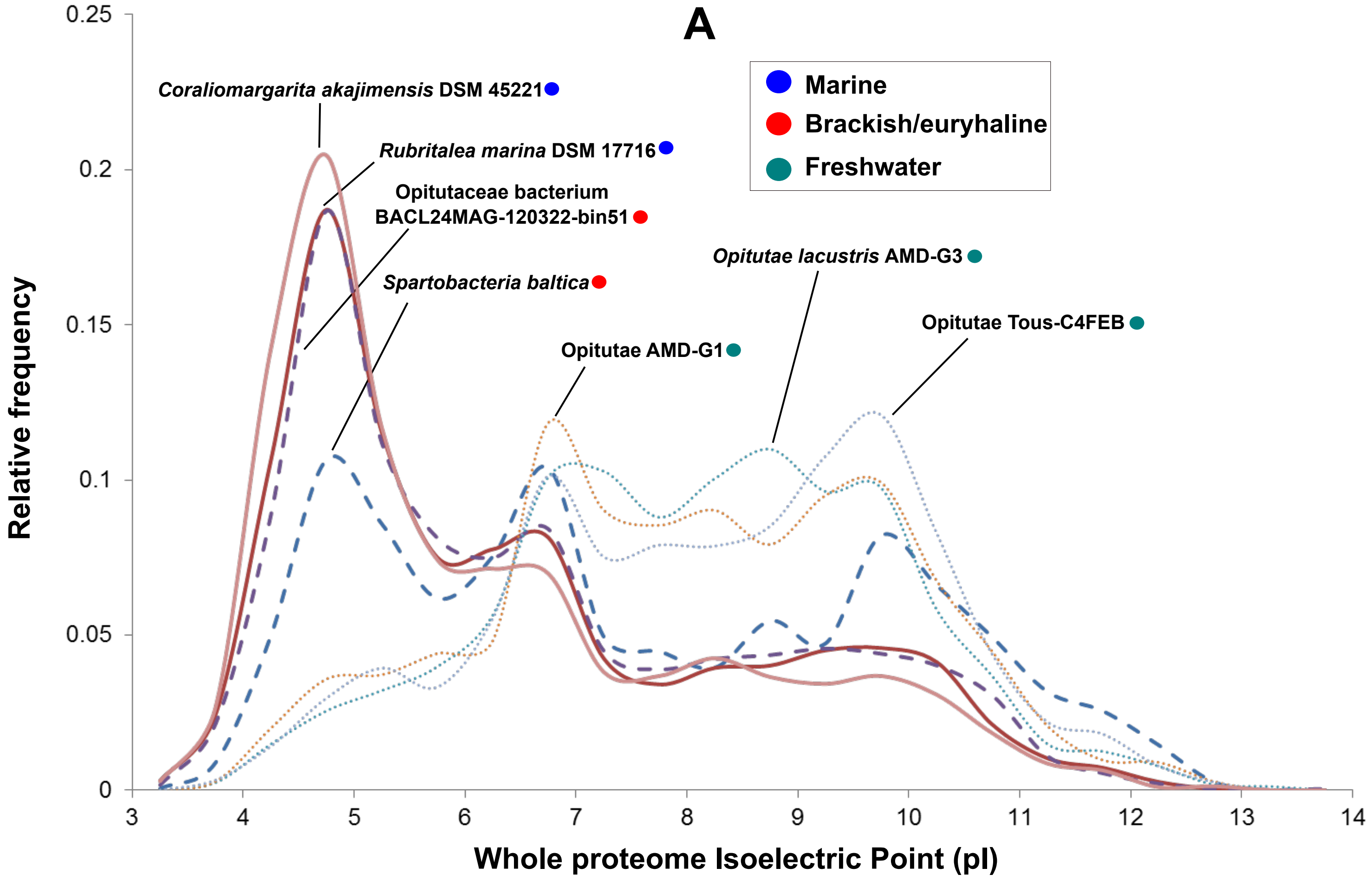

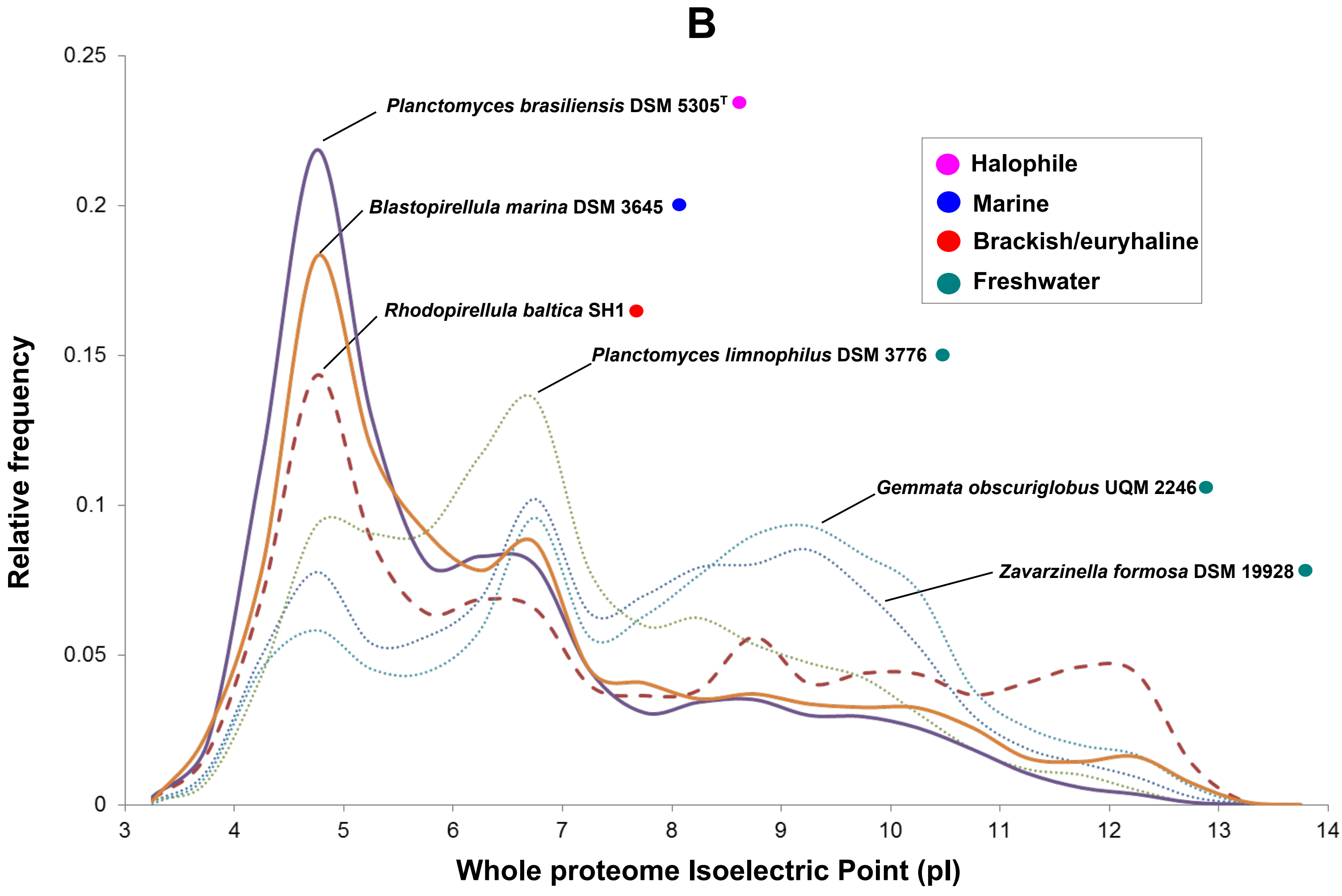

Figure S5. Whole proteome pI versus relative frequency plot of some representatives from different habitats of A) phylum Verrucomicrobia and B) phylum Planctomycetes.

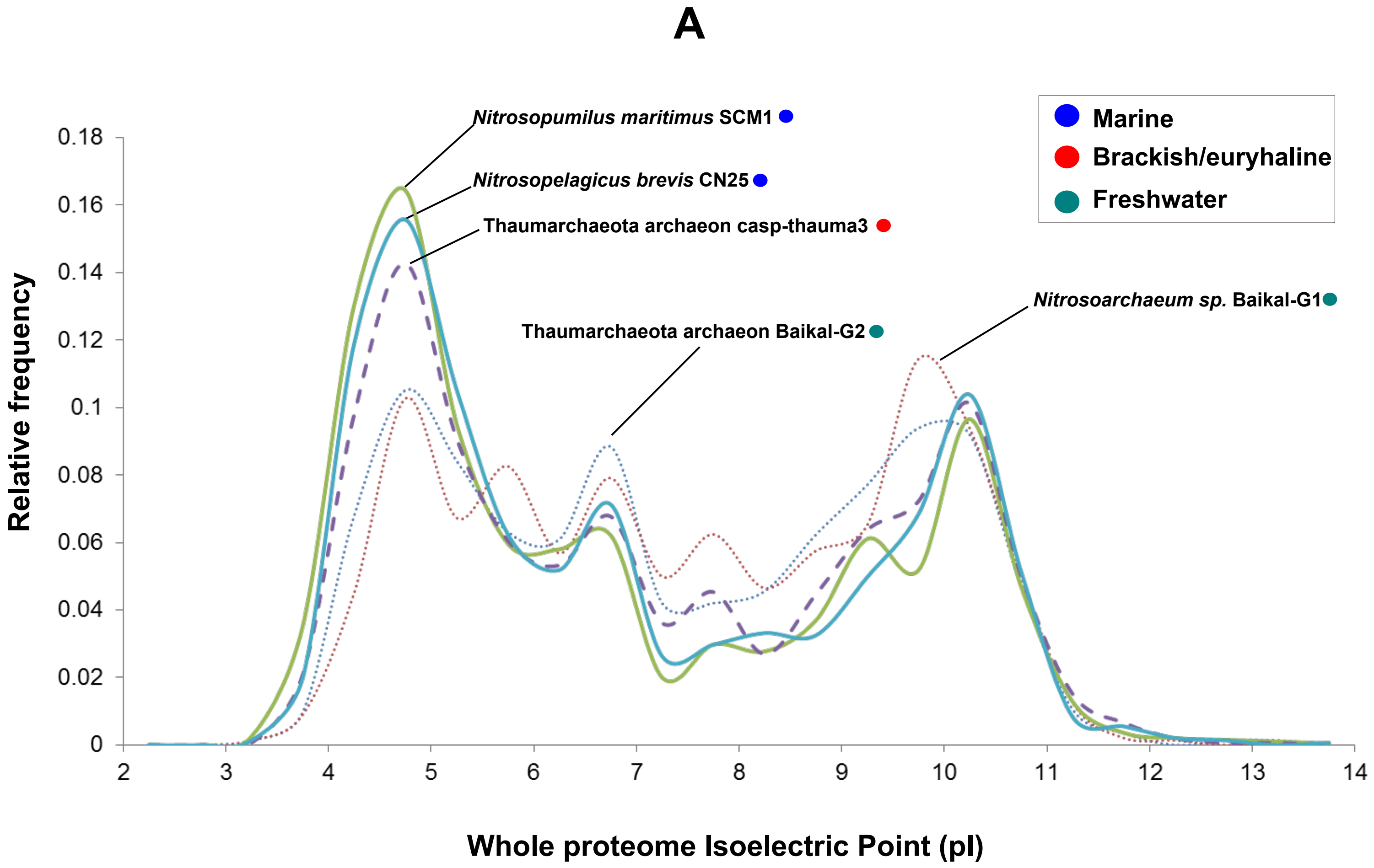

Figure S6. Whole proteome pI versus relative frequency plot of some representatives from different habitats of the phylum Thaumarchaeota.

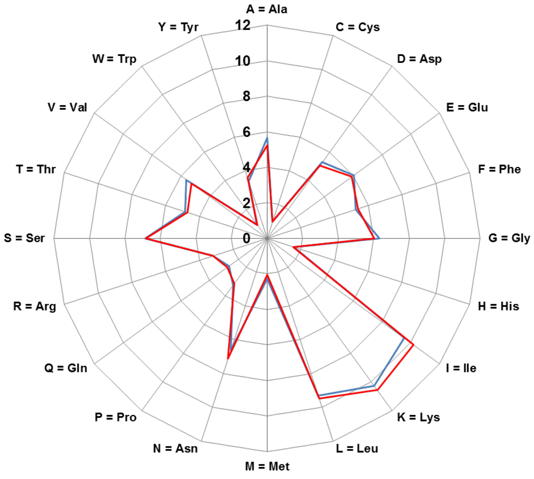

Pelagibacteraceae Baikal-G1

***Pelagibacter ubique* HTCC 7214**

Property	Residues	P.ubique HTCC 7214 (Mole %)	Pelagibacteraceae Baikal-G1 (Mole %)
Tiny	(A+C+G+S+T)	24.689	23.791
Small	(A+B+C+D+G+N+P+S+T+V)	45.48	44.387
Aliphatic	(A+I+L+V)	30.134	30.152
Aromatic	(F+H+W+Y)	11.187	11.433
Non-polar	(A+C+F+G+I+L+M+P+V+W+Y)	52.627	52.274
Polar	(D+E+H+K+N+Q+R+S+T+Z)	47.373	47.726
Charged	(B+D+E+H+K+R+Z)	26.407	26.294
Basic	(H+K+R)	15.069	15.327
Acidic	(B+D+E+Z)	11.338	10.967

Amino acid	*P.ubique* HTCC 7214 (Mole %)	Pelagibacteraceae Baikal-G1 (Mole %)
A = Ala	5.652	5.221
C = Cys	1.001	0.989
D = Asp	5.302	5.07
E = Glu	6.036	5.897
F = Phe	5.252	5.398
G = Gly	6.33	6.035
H = His	1.593	1.555
I = Ile	9.552	10.191
K = Lys	10.28	10.565
L = Leu	9.317	9.472
M = Met	2.291	2.069
N = Asn	6.599	7.107
P = Pro	3.277	3.151
Q = Gln	2.66	2.78
R = Arg	3.196	3.208
S = Ser	6.869	6.817
T = Thr	4.838	4.728
V = Val	5.613	5.268
W = Trp	0.968	0.905
Y = Tyr	3.374	3.575

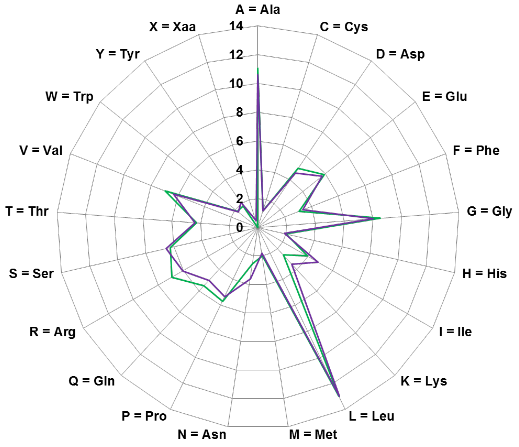

***Synechococcus lacustris* Tous**

***Synechococcus* sp. RCC307**

Property	Residues	*Synechococcus* sp. RCC 307 (Mole %)	*Synechococcus lacustris* Tous (Mole %)
Tiny	(A+C+G+S+T)	31.323	30.845
Small	(A+B+C+D+G+N+P+S+T+V)	51.438	50.679
Aliphatic	(A+I+L+V)	34.943	34.77
Aromatic	(F+H+W+Y)	8.7	9.042
Non-polar	(A+C+F+G+I+L+M+P+V+W+Y)	59.039	58.322
Polar	(D+E+H+K+N+Q+R+S+T+Z)	40.961	41.204
Charged	(B+D+E+H+K+R+Z)	22.394	21.638
Basic	(H+K+R)	11.53	11.363
Acidic	(B+D+E+Z)	10.865	10.275

Amino acid	*Synechococcus* sp. RCC 307	*Synechococcus lacustris* Tous
A = Ala	11.07	10.624
C = Cys	1.219	1.222
D = Asp	4.959	4.592
E = Glu	5.906	5.683
F = Phe	3.057	3.325
G = Gly	8.509	8.043
H = His	2.016	1.873
I = Ile	3.953	4.796
K = Lys	2.61	3.463
L = Leu	12.985	13.017
M = Met	1.992	1.802
N = Asn	2.527	3.607
P = Pro	5.692	5.316
Q = Gln	5.513	5.003
R = Arg	6.904	6.027
S = Ser	6.244	6.546
T = Thr	4.282	4.41
V = Val	6.936	6.319
W = Trp	1.814	1.749
Y = Tyr	1.813	2.094
X = Xaa	0	0.474

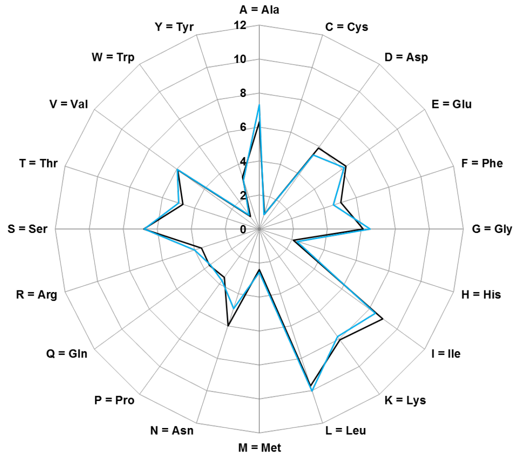

***Methylopumilus planktonicus* MMS-2-53**

Methylophilales bacterium MBRS-H7

Property	Residues	Methylophilales bacterium MBRSH7 (Mole%)	*Methylopumilus planktonicus* MMS-2-53 (Mole%)
Tiny	(A+C+G+S+T)	24.848	26.443
Small	(A+B+C+D+G+N+P+S+T+V)	46.146	46.476
Aliphatic	(A+I+L+V)	30.884	31.73
Aromatic	(F+H+W+Y)	11.257	10.982
Non-polar	(A+C+F+G+I+L+M+P+V+W+Y)	52.989	54.054
Polar	(D+E+H+K+N+Q+R+S+T+Z)	47.011	45.946
Charged	(B+D+E+H+K+R+Z)	25.896	25.722
Basic	(H+K+R)	13.716	14.161
Acidic	(B+D+E+Z)	12.18	11.561

Amino acid	Methylophilales bacterium MBRSH7	*Methylopumilus planktonicus* MMS-2-53
A = Ala	6.294	7.302
C = Cys	0.92	0.887
D = Asp	5.891	5.396
E = Glu	6.289	6.166
F = Phe	5.047	4.587
G = Gly	6.117	6.506
H = His	2.085	2.341
I = Ile	8.965	8.437
K = Lys	8.045	7.824
L = Leu	9.695	10.021
M = Met	2.393	2.538
N = Asn	5.974	4.915
P = Pro	3.503	3.752
Q = Gln	3.624	3.561
R = Arg	3.587	3.996
S = Ser	6.805	6.758
T = Thr	4.713	4.99
V = Val	5.929	5.97
W = Trp	0.91	1.024
Y = Tyr	3.215	3.029

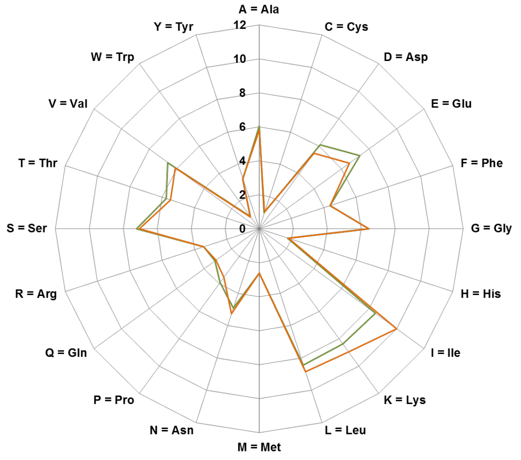

***Nitrosoarchaeum* sp. Baikal-G1**

***Nitrosopumilus maritimus* SCM1**

Property	Residues	*Nitrosopumilus maritimus* SCM1	*Nitrosoarchaeum* sp. Baikal-G1
Tiny	(A+C+G+S+T)	26.42	25.853
Small	(A+B+C+D+G+N+P+S+T+V)	47.982	46.256
Aliphatic	(A+I+L+V)	29.586	30.748
Aromatic	(F+H+W+Y)	10.147	10.168
Non-polar	(A+C+F+G+I+L+M+P+V+W+Y)	51.899	52.774
Polar	(D+E+H+K+N+Q+R+S+T+Z)	48.101	47.218
Charged	(B+D+E+H+K+R+Z)	27.047	26.295
Basic	(H+K+R)	13.586	14.204
Acidic	(B+D+E+Z)	13.461	12.091

Amino acid	*Nitrosopumilus maritimus* SCM1	*Nitrosoarchaeum* sp. Baikal-G1
A = Ala	6.055	5.792
C = Cys	0.983	1.056
D = Asp	6.124	5.509
E = Glu	7.337	6.582
F = Phe	4.403	4.382
G = Gly	6.445	6.47
H = His	1.772	1.824
I = Ile	8.461	10.006
K = Lys	8.393	8.952
L = Leu	8.432	8.855
M = Met	2.606	2.611
N = Asn	4.896	5.255
P = Pro	3.904	3.544
Q = Gln	3.222	3.133
R = Arg	3.42	3.428
S = Ser	7.199	7.05
T = Thr	5.738	5.485
V = Val	6.638	6.095
W = Trp	0.894	0.855
Y = Tyr	3.078	3.108

Figure S7. Star diagrams and amino acid composition of prokaryotic relatives from marine and freshwater origin.

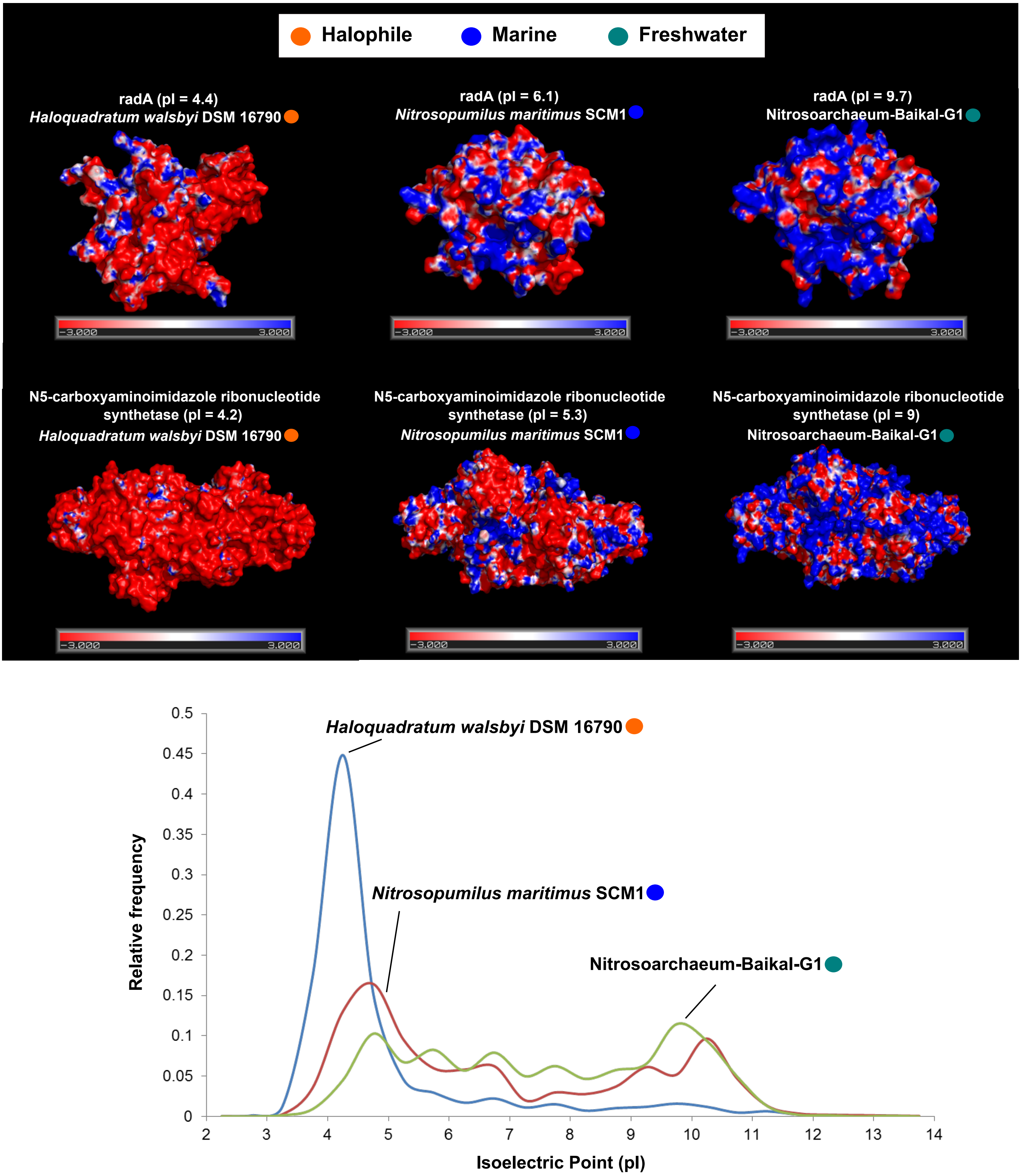

Figure S8. Structural model of proteins from different habitat-adapted archaea. Insets show electrostatic surface potential 3D models of N5-carboxyaminoimidazole ribonucleotide synthetase (secreted protein) and radA (cytoplasmic). The potentials were coloured from -3 kcal mol-1(red) to +3 kcal mol-1 (blue). Whole proteome pI versus relative frequency plot of *Haloquadratum walsbyi* DSM 16790 (halophile, culture), *Nitrosopumilus maritimus* SCM1 (marine, culture), *Nitrosoarchaeum* sp. Baikal-G1 (freshwater, MAG).

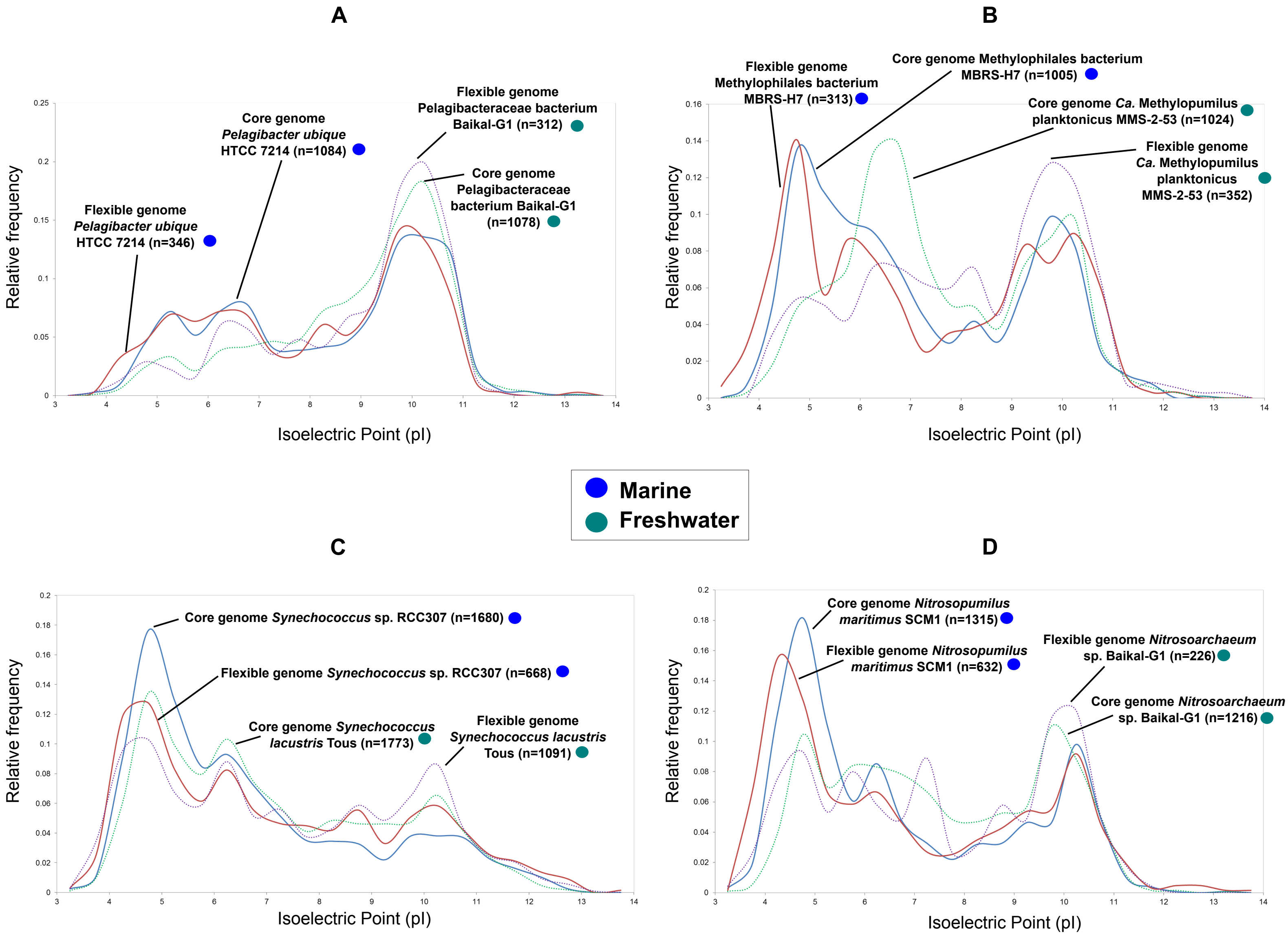

Figure S9. Isoelectric point versus relative frequency plot of the pan-genome (core and flexible genome) of freshwater and marine prokaryotes. N indicates the number of proteins in either core or flexible genomes. A) *P.ubique* HTCC 7214 and Pelagibacteraceae bacterium Baikal-G1. B) *Ca*. Methylopumilus planktonicus MMS-2-53 and Methylophilales bacterium MBRS-H7. C) *Synechococcus* sp. RCC307 and *Synechococcus lacustris* Tous. D) *Nitrosopumilus maritimus* SCM1 and *Nitrosoarchaeum* sp. Baikal-G1.

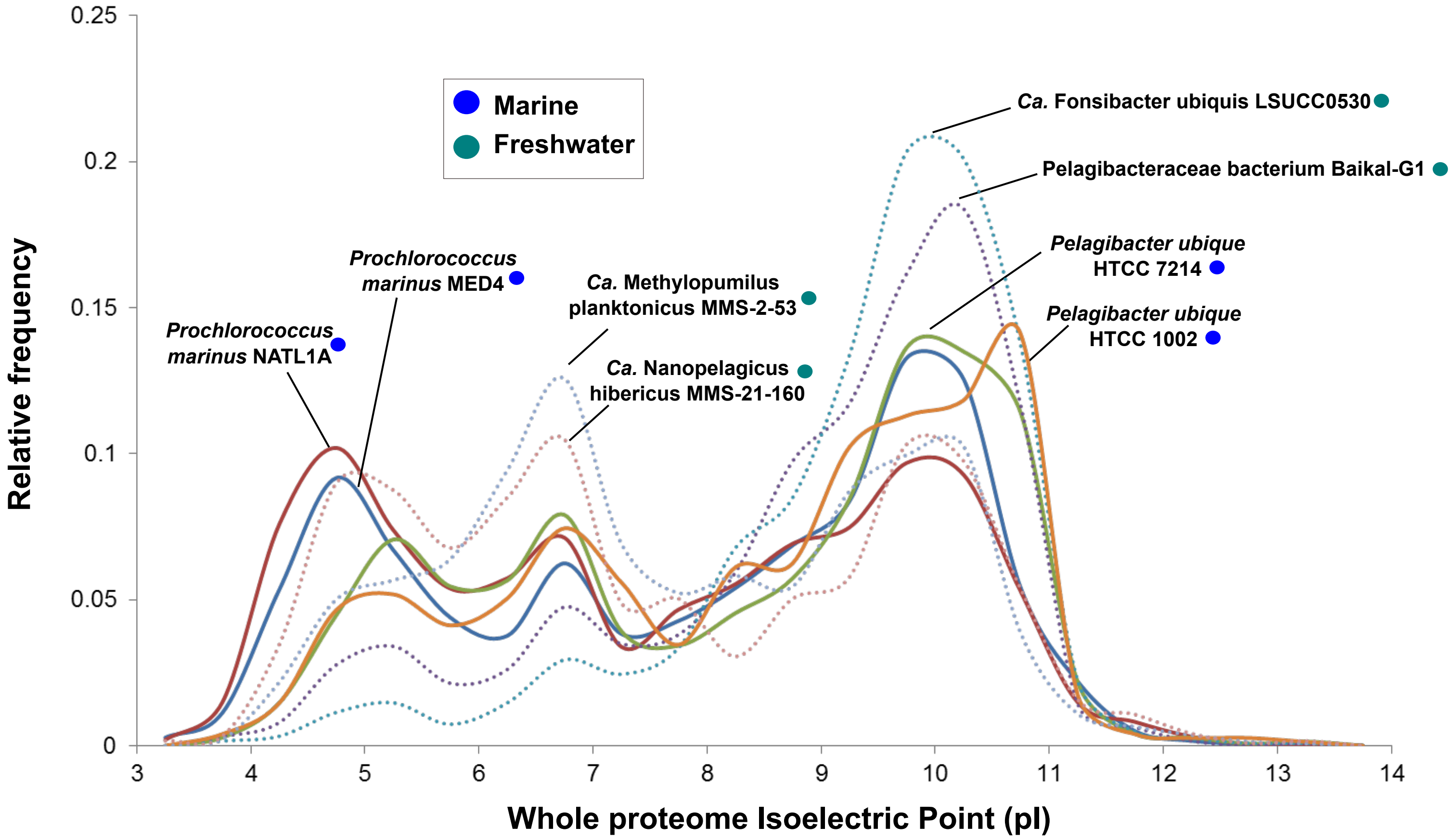

Figure S10. Whole proteome pI versus relative frequency plot of some streamlined bacteria from different habitats.
